# The chemical composition of secondary organic aerosols regulates transcriptomic and metabolomic signaling in an epithelial-endothelial in vitro coculture

**DOI:** 10.1186/s12989-024-00600-x

**Published:** 2024-09-19

**Authors:** Svenja Offer, Sebastiano Di Bucchianico, Hendryk Czech, Michal Pardo, Jana Pantzke, Christoph Bisig, Eric Schneider, Stefanie Bauer, Elias J. Zimmermann, Sebastian Oeder, Elena Hartner, Thomas Gröger, Rasha Alsaleh, Christian Kersch, Till Ziehm, Thorsten Hohaus, Christopher P. Rüger, Simone Schmitz-Spanke, Jürgen Schnelle-Kreis, Martin Sklorz, Astrid Kiendler-Scharr, Yinon Rudich, Ralf Zimmermann

**Affiliations:** 1 Joint Mass Spectrometry Center (JMSC) at Comprehensive Molecular Analytics (CMA), Helmholtz Zentrum München, Ingolstädter Landstr. 1, D-85764 Neuherberg, Germany; 2 Joint Mass Spectrometry Center (JMSC) at Analytical Chemistry, Institute of Chemistry, University of Rostock, Albert-Einstein-Str. 27, D-18059 Rostock, Germany; 3 Department Life, Light & Matter (LLM), University of Rostock, D-18051 Rostock, Germany; 4 Department of Earth and Planetary Sciences, Faculty of Chemistry, Weizmann Institute of Science, 234 Herzl Street, POB 26, Rehovot, ISR-7610001 Israel; 5 Institute and Outpatient Clinic of Occupational, Social and Environmental Medicine, Friedrich-Alexander University of Erlangen-Nuremberg, Henkestr. 9-11, D-91054 Erlangen, Germany; 6 Institute of Energy and Climate Research, Forschungszentrum Jülich GmbH, Troposphere (IEK-8), Wilhelm- Johen-Str, D-52428 Jülich, Germany

**Keywords:** Epithelial-endothelial coculture, Secondary organic aerosols (SOA), Airway remodeling, Inflammation, Endothelial dysfunction

## Abstract

**Background:**

The formation of secondary organic aerosols (SOA) by atmospheric oxidation reactions substantially contributes to the burden of fine particulate matter (PM_2.5_), which has been associated with adverse health effects (e.g., cardiovascular diseases). However, the molecular and cellular effects of atmospheric aging on aerosol toxicity have not been fully elucidated, especially in model systems that enable cell-to-cell signaling.

**Methods:**

In this study, we aimed to elucidate the complexity of atmospheric aerosol toxicology by exposing a coculture model system consisting of an alveolar (A549) and an endothelial (EA.hy926) cell line seeded in a 3D orientation at the air‒liquid interface for 4 h to model aerosols. Simulation of atmospheric aging was performed on volatile biogenic (β-pinene) or anthropogenic (naphthalene) precursors of SOA condensing on soot particles. The similar physical properties for both SOA, but distinct differences in chemical composition (e.g., aromatic compounds, oxidation state, unsaturated carbonyls) enabled to determine specifically induced toxic effects of SOA.

**Results:**

In A549 cells, exposure to naphthalene-derived SOA induced stress-related airway remodeling and an early type I immune response to a greater extent. Transcriptomic analysis of EA.hy926 cells not directly exposed to aerosol and integration with metabolome data indicated generalized systemic effects resulting from the activation of early response genes and the involvement of cardiovascular disease (CVD) -related pathways, such as the intracellular signal transduction pathway (PI3K/AKT) and pathways associated with endothelial dysfunction (iNOS; PDGF). Greater induction following anthropogenic SOA exposure might be causative for the observed secondary genotoxicity.

**Conclusion:**

Our findings revealed that the specific effects of SOA on directly exposed epithelial cells are highly dependent on the chemical identity, whereas non directly exposed endothelial cells exhibit more generalized systemic effects with the activation of early stress response genes and the involvement of CVD-related pathways. However, a greater correlation was made between the exposure to the anthropogenic SOA compared to the biogenic SOA. In summary, our study highlights the importance of chemical aerosol composition and the use of cell systems with cell-to-cell interplay on toxicological outcomes.

**Graphical Abstract:**

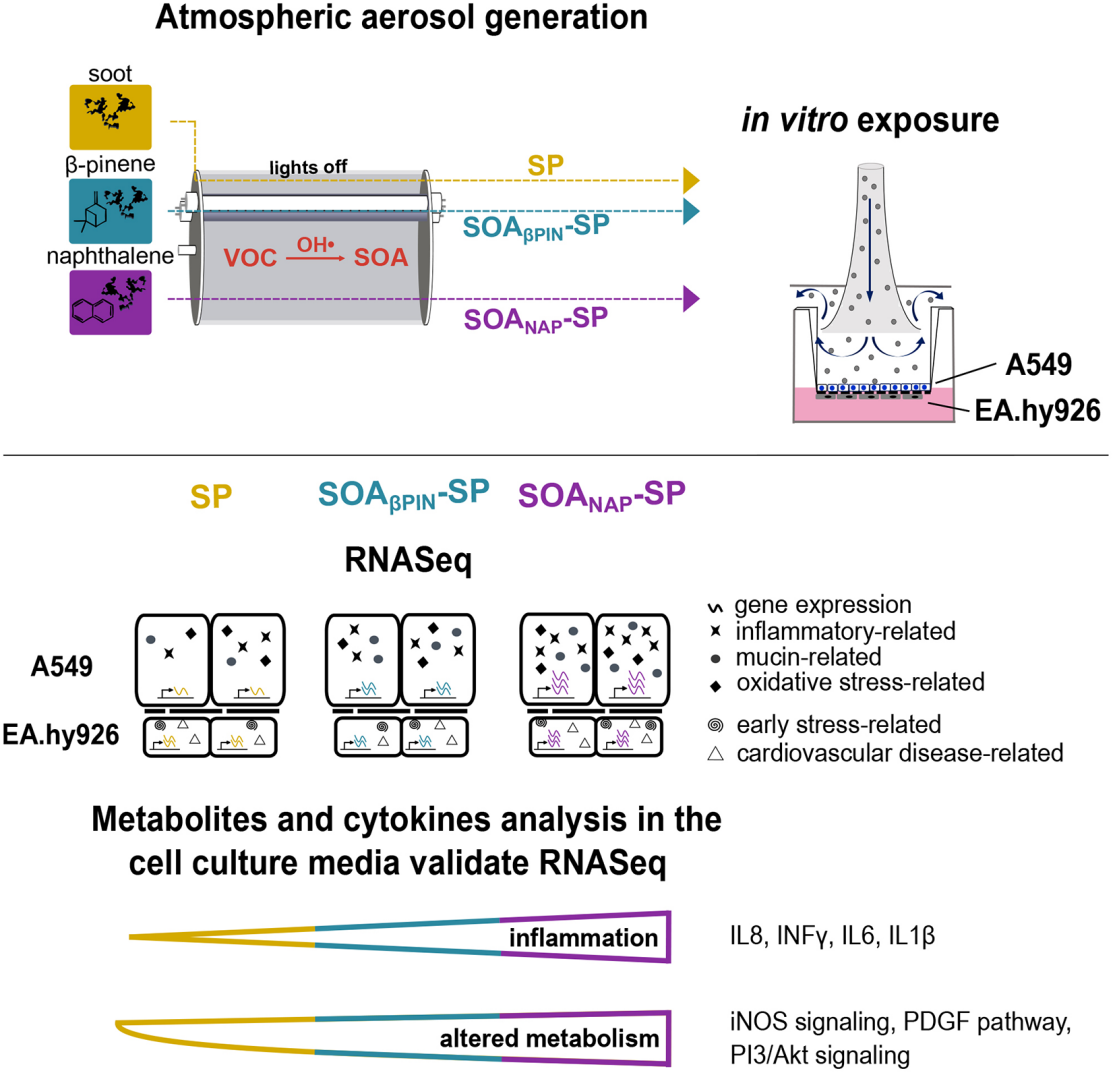

**Supplementary Information:**

The online version contains supplementary material available at 10.1186/s12989-024-00600-x.

## Background

Air pollution by aerosols is a complex mixture of compounds in the gaseous and particulate phases. It is well established that outdoor air pollution has adverse health outcomes, resulting in high levels of mortality and morbidity worldwide [1–3]. Multiple studies have shown a strong correlation between exposure to ambient fine particulate matter (PM_2.5_) and the occurrence of respiratory and cardiovascular diseases (CVD). Due to its size, PM_2.5_ can penetrate and remain deep in the lungs and induce direct pulmonary effects, as well as effects on other organs or systemic effects, such as those in the cardiovascular system [4, 5]. A cohort study of healthy individuals revealed endothelial cell dysfunction as a key player in the induction of CVD after PM_2.5_ inhalation [6]. Supporting evidence was also found in the in vivo experiments by Davel, Lemos [7] and Tamagawa, Bai [8] who demonstrated vascular endothelial dysfunction after PM exposure.

A major fraction of ambient PM is, however, composed of secondary organic aerosols (SOA), generated from low volatility products of atmospheric oxidation reactions of gaseous anthropogenic or biogenic organic compounds [9]. SOA formation can occur either through nucleation and growth of new molecular clusters [10] or condensation of oxidized vapors on preexisting particles [e.g., soot particles (SP)] [11, 12]. The composition of SOA includes a vast range of organic compounds, which are functionalized with moieties of particularly carboxylic acids, carbonyls and alcohols. However, the individual SOA composition varies upon SOA precursor and atmospheric aging conditions [13, 14]. Previous studies investigated several biological endpoints in in vitro exposures or the oxidative potential of SOA in acellular assays from combustion aerosols, such as from biomass burning or road traffic, or SOA precursors of biogenic or anthropogenic origin [15]. Recently, exposure to SOA has been shown to contribute substantially to mortality associated with air pollution [16]. Pye, Ward-Caviness [17] demonstrated a correlation between annual average SOA concentrations and county-level cardiorespiratory death rates. The importance of understanding the relationship between aerosol composition and adverse biological effects for determining the toxicity mechanisms of different SOA has been demonstrated in studies with human airway cell lines [18–21] and lung lining fluid [22]; however, this topic elusive in model systems that enable cell-to-cell signaling.

In this follow-up work, we aimed to elucidate the complexity of atmospheric aerosol toxicology by applying controlled model aerosols for realistic in vitro exposure at the air-liquid interface (ALI) by considering cell-to-cell signaling possibly driving systemic adverse effects. To achieve this goal, we have exposed a coculture model system consisting of a lung epithelial cell line (A549) and an endothelial cell line (EA.hy926) in a 3D orientation. A549 cell line is a widely used model cell line for nanoparticle toxicological studies, particularly concerning lung metabolism and carcinogenesis. When cultured at the ALI, it has been shown that A549 cells can express important characteristics of alveolar epithelial type II cells that are essential for the defense of inhaled toxicants (e.g., tight epithelial phenotype, surfactant production, detoxification by metabolic enzymes) [23]. To mimic the blood-air barrier as well as to investigate the interaction between the cells, we seeded EA.hy926 cells on the basolateral side of the insert membrane. This coculture model system was exposed to different dilutions (undiluted, 1:3; 1:10 or 1:30) of either an anthropogenic SOA from an aromatic precursor (naphthalene), mixed with SP (SOA_NAP_-SP), or a biogenic SOA from an aliphatic precursor (β-pinene) mixed with SP (SOA_βPIN_-SP). Comparisons were made with fresh SP. Recently, we were able to detect secondary genotoxicity and increased angiogenic potential in endothelial cells only after exposure to SOA_NAP_-SP [21], thus we hypothesized that non directly exposed endothelial cells may be differentially affected by the type of SOA. Therefore, we compared functional, transcriptional, and metabolic changes in the coculture model system after the exposure to SP, SOA_βPIN_-SP or SOA_NAP_-SP at the ALI system. An aerosol dilution of 1:3 was the focus of the study as we previously observed low toxic results and detected DNA breaks in the non-directly exposed EA.hy926 cells [21]. A549 and EA.hy926 cells were separated by an insert membrane, which enabled us to trace their specific transcriptomic changes. Moreover, secreted mediators, including cytokines and metabolites found in the sampled media, were used to validate the observed transcriptomic outcomes and to draw possible conclusions on the type-specific molecular effects of SOA.

## Results and discussion

### Experimental set-up

We have previously demonstrated that the exposure of our coculture model system (A549/EA.hy926 cells) to SOA_NAP_-SP revealed greater toxic effects than SOA_βPIN_-SP and pure SP. Calculated cellular deposition of the aerosol dilutions (undiluted, 1:3, 1:10 and 1:30) ranged from 0.9 ng/cm^2^ (1:30 dilution) to 28 ng/cm^2^ (undiluted) for SOA_NAP_-SP and from 0.6 ng/cm^2^ (1:30 dilution) to 17 ng/cm^2^ (undiluted) for SOA_βPIN_-SP. According to calculations made by Paur, Cassee [24] for in vitro nanotoxicology studies, a cellular deposition of 0.75 ng/cm^2^ is suggested for a realistic ambient exposure up to 130 ng/cm^2^ for worst-case occupational exposure which corresponds to a daily dose Therefore, our estimated cellular deposition is covering different exposure conditions ranging from ambient to mild occupational settings [21, 24]. In addition, the exposure time was set at 4 h to obtain first indications of acute stress responses of the cells. At the functional level, we observed that SOA_NAP_-SP augmented the secretion of lactate dehydrogenase (LDH), malondialdehyde (MDA) and interleukin 8 (IL8) as signs for cytotoxicity, oxidative stress, and inflammation. Moreover, we detected secondary genotoxicity and a greater angiogenic potential in EA.hy926 cells exposed to SOA_NAP_-SP, suggesting a possible activation of the non-directly exposed endothelial cells in the coculture system. Table S1 summarizes previously published results [21]. In this work we elucidate the transcriptional and metabolic changes in our coculture model system after SOA exposure and focused on aerosol dilution 1:3 that corresponds to low toxic exposures [21].

### Characterization of the generated SOA revealed similar physical but distinct chemical properties

To investigate the role of SOA to influence cellular mechanisms, we took advantage of previously described in-depth characterization of the two models of SOA [21]. Briefly, the aging of both SOA precursors (biogenic: β-pinene and anthropogenic: naphthalene) together with soot particles was conducted under the same OH exposure, which is equivalent to approximately 3 days of atmospheric photochemical aging. Using pure CASTs soot as primary particles, a substantial fraction of the photooxidation products of β-pinene and naphthalene condensed on the SP, resulting in SOA_βPIN_-SP and SOA_NAP_-SP. Consequently, the particle size distributions and the particle diameters (means ∼ 115 nm) of the two SOA were remarkably similar. However, there were differences in the chemical properties of the aerosol particles and the gas phase.

**Fig. 1:**
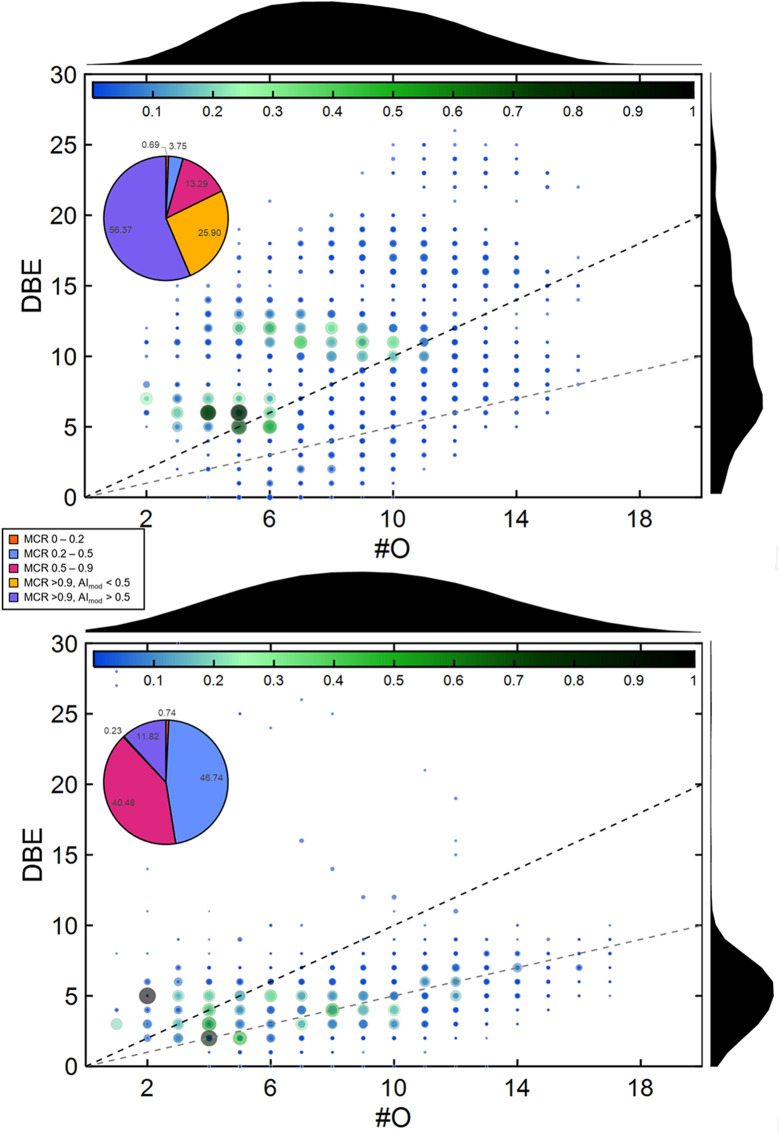
Maximum carbonyl ratio (MCR) and aromaticity of SOA_NAP_-SP and SOA_βPIN_-SP. Number of oxygen atoms (#O) vs. double bond equivalents (DBE) in SOA_NAP_-SP (top) and SOA_βPIN_-SP (bottom) with pie charts depicting the percentage of peaks in individual bins of the maximum carbonyl ratio (MCR) and aromaticity index (AI) for ESI in positive mode

Particularly in the semi-volatile fraction of the PM, which was investigated by two-dimensional gas chromatography-mass spectrometry, the chemical base structure of the SOA precursors was maintained. While SOA_βPIN_-SP contained aliphatic and cyclic SOA species, functionalized aromatic compounds were detected in SOA_NAP_-SP. As two-dimensional gas chromatography-mass spectrometry is limited to semi-volatile compounds in aerosol particles, we investigated the abundance of this compound class via direct-infusion ultrahigh resolution mass spectrometry with the concept of the maximum carbonyl ratio (MCR) [25]. According to the electrospray ionization in positive mode (+ ESI) results for the two SOA-SP, a similar range of #O is covered, but the #O distribution in SOA_βPIN_-SP is more shifted to a larger #O than that in SOA_NAP_-SP. In contrast, the DBE in SOA_NAP_-SP has a multimodal distribution and correlates with the detection of dimers and trimers to larger oligomers [20], whereas the DBE in SOA_βPIN_-SP has a single mode at 5 and does not significantly exceed a DBE of 10. These molecular properties allow us to conclude that for SOA_NAP_-SP > 50% of the peak intensity corresponds to oxygenated (O-containing) compounds belonging to unsaturated carbonyls, while for SOA_βPIN_-SP, the percentage is only ≈ 10% (Fig. 1). As a complementary ionization technique, ESI in negative mode (-ESI) is capable of detecting compounds with acidic functional groups, such as carboxylic acids, as well as 1,3-dicarbonyls and other derivatives with acidic hydrogen. A similar observation can be made in the -ESI results, but with a less distinct pattern for oligomeric structures (Figure S1). Moreover, the percentage of compounds with MCR ≥ 1 was lower than that with + ESI for both aerosols, revealing that a substantial number of carbonyl moieties with unsaturated carbon backbones are carboxyl groups or conjugated carbonyls.

To better understand the gas phase composition, we investigated the differences in the molecular composition by proton-transfer-reaction (PTR) high-resolution time-of-flight mass spectrometry (PTR-TOFMS; Table 1 and S2). Small molecules with carbon numbers ranging from one (e.g., formaldehyde) to ten (e.g., pinonaldehyde) had higher oxygen contents for volatile organic compounds (VOC) in SOA_NAP_-SP (CHO_1_: 137 ± 12 ppb; CHO_*n*>1_: 173 ± 13 ppb) than in SOA_βPIN_-SP (CHO_1_: 336 ± 26 ppb; CHO_*n*>1_: 130 ± 13 ppb). However, oxygenated VOCs were overall more abundant in SOA_βPIN_-SP than in the other samples, which may be explained by the oxidation mechanism. In the OH-initiated photooxidation of β-pinene, acetone is one of the major first-generation oxidation products [26] and the most abundant individual VOC of SOA_βPIN_-SP in our study, followed by other small C1 and C2 species, i.e., formaldehyde (CH_2_O), methanol (CH_4_O), formic acid (CH_2_O_2_) and acetic acid (CH_4_O_2_).

**Table 1:** Chemical properties of the gas-phase of atmospheric photooxidation (aging) by OH radicals in a PAM (potential aerosol mass) reactor of SP (CAST soot; 1 mg/m^3^) together with either naphthalene (4 mg/m^3^) or β-pinene (4 mg/m^3^), forming SOA_NAP_-SP and SOA_βPIN_-SP, respectively, measured by proton-transfer-reaction (PTR) high-resolution time-of-flight mass spectrometry PTR-TOFMS. The results are shown as the mean ± SD of *n* = 4 independent experiments

Compound class	SOA_NAP_-SP	SOA_βPIN_-SP
	mean ± SD [ppb]	mean ± SD [ppb]
CH	116 ± 88	94 ± 5
CHO_n_*n* = 1	137 ± 12	336 ± 26
CHO_*n*>2_	173 ± 13	130 ± 13
CHN + CHNO	52 ± 1	56 ± 2

In total, these five most abundant VOC accounted for 55% of the total VOC detected by PTR-TOFMS at SOA_βPIN_-SP. Generally, CH and N-containing VOC species originated from primary emissions of CAST or from unreacted SOA precursors, which was particularly the case for SOA_NAP_-SP. Here, naphthalene exceeded the concentration of all the other detected VOC and accounted for 53% of the total CH species. The other abundant individual VOC for SOA_NAP_-SP included an unknown species with the sum of the formula C_5_H_2_O_2_, methanol (CH_4_O), butene (C_4_H_8_) and formic acid (CH_2_O_2_). Despite the distinct differences in VOC concentration compared to that of SOA_βPIN_-SP, for SOA_NAP_-SP, the five most abundant VOC accounted for 57% of the total detected VOCs. Taken together, the key findings over the entire molecular range were the prevailing aromaticity of the naphthalene precursor and its greater tendency to form unsaturated, highly oxygenated species compared to those of the β-pinene precursor.

### ALI exposure to SOA_NAP_-SP induced the strongest changes in both epithelial (A549) and endothelial (EA.hy926) human lung cells

**Fig. 2:**
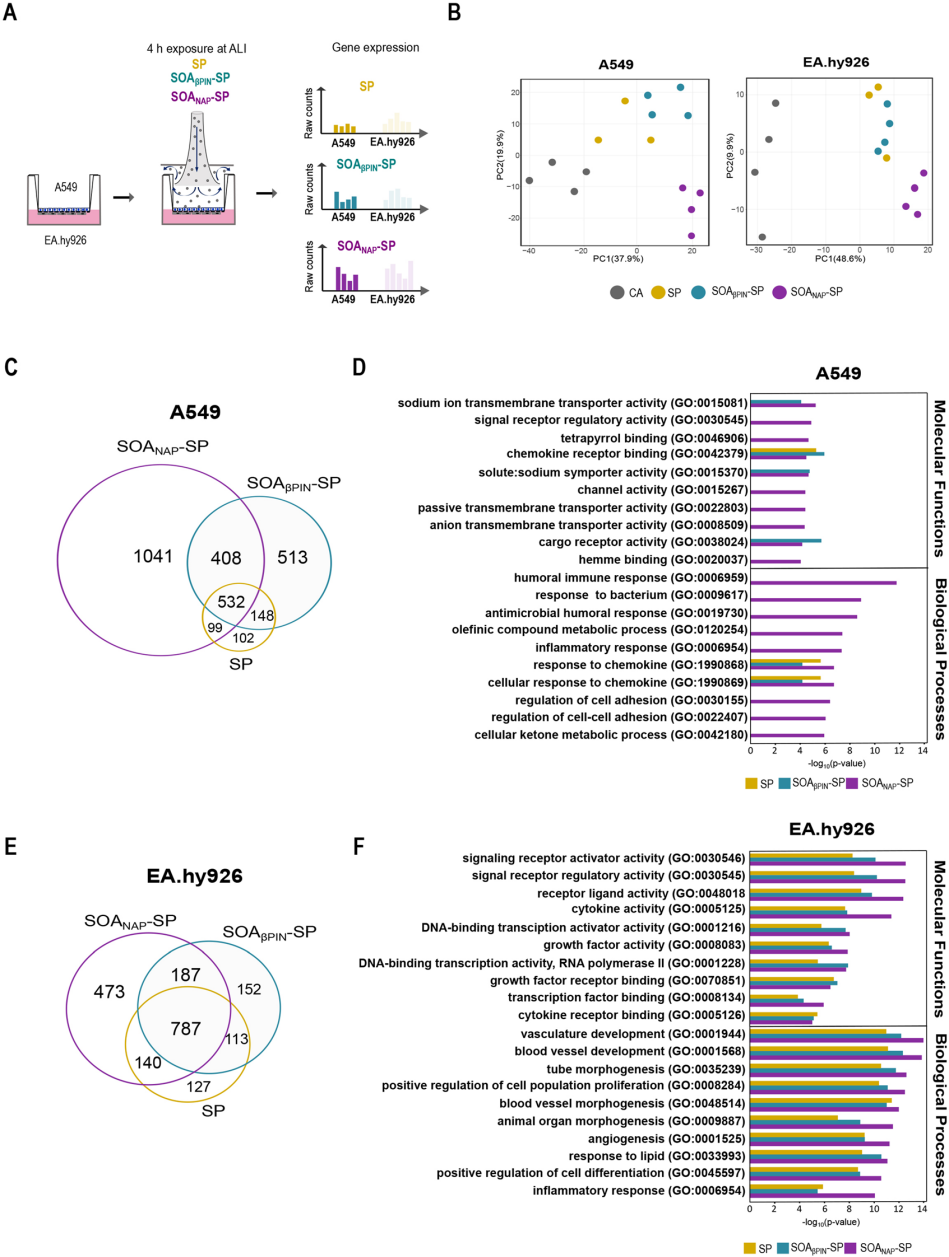
Transcriptional analysis of aerosol-induced effects in A549 and EA.hy926 cells. (**A**) Schematic representation of the experimental setup. A coculture model system consisting of A549 lung epithelial cells on one side of the insert membrane and EA.hy926 endothelial cells on the other side were exposed for 4 h at the air-liquid interface (ALI) to two SOA, namely, SOA_βPIN_-SP and SOA_NAP_-SP and SP. Transcriptional changes in A549 and EA.hy926 cells were also analyzed. (**B**) Principal component analysis (PCA) of all genes with batch correction and normalization to colors based on exposure to clean air (CA), SOA_NAP_-SP, and SOA_βPIN_-SP; *n* = 4 independent experiments and SP of *n* = 3 experiments of A549 and EA.hy926 cells. (**C**) Venn diagram of the unique and overlapping genes associated with the up- and downregulated genes (adjusted p-value ≤ 0.05, and log_2_FC ≥ 0.5 and log_2_FC ≤ -0.5) in A549 cells after exposure to SP, SOA_βPIN_-SP or SOA_NAP_-SP compared to the CA control. (**D**) Gene Ontology (GO) analysis of differentially expressed genes (DEGs) related to biological processes and molecular functions between A549 cells exposed to different aerosols (SP, SOA_βPIN_-SP, and SOA_NAP_-SP) and the CA Ctrl. (**E** and **F**) Same as in (**C** and **D**) but based on the analysis of EA.hy926 cells

As a result of the generated physically similar but chemically distinct SOA, we are now able to assess how different chemistries impact cell effects. To identify the molecular mechanisms induced by an anthropogenic (SOA_NAP_-SP) and a biogenic (SOA_βPIN_-SP) model of SOA, as well as by SP alone, in our coculture system, the directly exposed A549 cells and the non-directly exposed EA.hy926 cells were separately harvested after 4 h to perform RNA-Seq (Fig. 2A). The results showed distinct gene expression patterns in A549 and EA.hy926 cells exposed to all aerosols (both SOA and SP) and to the clean air (CA) control (Fig. 2B and S2A). Next, we conducted differential gene expression analysis between cells exposed to SOA_βPIN_-SP, SOA_NAP_-SP, or SP and those exposed to CA control. In A549 cells, we found 881 DEGs (298 genes upregulated and 583 genes downregulated) after SP exposure (Figure S2B and S3A), 1601 DEGs (532 genes upregulated and 1069 genes downregulated) after SOA_βPIN_-SP exposure (Figure S2B and S3B) and 2080 DEGs (805 upregulated and 1275 downregulated) after SOA_NAP_-SP exposure (Figure S2B and S3C), indicating the differential impact of exposure both in terms of the number of regulated genes and the overall patterns of up- and downregulation, which varied from approximately 33–34% of upregulated DEGs following SP and SOA_βPIN_-SP exposure to 39% after SOA_NAP_-SP treatment. The most up- and downregulated genes in A549 cells after the exposure to SP, SOA_βPIN_-SP or SOA_NAP_-SP compared to those in the CA control are summarized in Tables S2, S3 and S4, respectively. Consistently, comparison of DEGs between SP, SOA_βPIN_-SP and SOA_NAP_-SP revealed the most uniquely expressed genes after exposure to SOA_NAP_-SP (50%) followed by SOA_βPIN_-SP (32%), whereas the exposure to SP showed the greatest overlap of DEGs (60%) between all three aerosol types in A549 cells (Fig. 2C). Gene Ontology (GO) analysis of the significantly upregulated genes in A549 cells revealed several enriched processes, such as “transmembrane transporter activity”, “receptor binding”, “immune responses”, and “metabolic processes”, especially for SOA_NAP_-SP (Fig. 2D). Notably, channel and transporter activity are known to play important roles in pulmonary homeostasis, and their dysregulation could contribute to pulmonary diseases [27]. Within those GO groups, we detected several genes whose expression was significantly upregulated after exposure to both SOA in our dataset (*MFSD2A*,* TRPC6* or *KCNK1*) that were recently correlated specifically with lung injury, airway inflammation and lung cancer [28–30]. Moreover, the importance of immunological and metabolic pathways activated in response to SOA exposure has already been noted at the protein level in monocultures of lung epithelial cells and murine macrophages [18, 31]. However, to our knowledge, a more in-depth characterization of the biological effects of direct and indirect exposure of cocultured cells is still lacking. By analyzing endothelial cells from the EA.hy926 cell line separately, we focused on cell-to-cell interactions with lung epithelial A549 cells. In EA.hy926 cells, we found 1167 DEGs (571 upregulated genes and 596 downregulated genes) after SP exposure (Figure S2B and S3D), 1239 DEGs (555 upregulated genes and 684 downregulated genes) after SOA_βPIN_-SP exposure (Figure S2B and S3E) and 1587 DEGs (674 upregulated genes and 913 downregulated genes) after SOA_NAP_-SP exposure (Figure S2B and S3E). This finding is consistent with the greater toxicogenomic effects that we observed in A549 cells after exposure to SOA_NAP_-SP. Interestingly, exposure to SOA_NAP_-SP induced greater imbalances between the up- and downregulated genes (42.5% and 57.5% respectively) that were not detected after exposure to SP (49% up- and 51% downregulation) or SOA_βPIN_-SP (45% up- and 55% downregulation). The most up- and downregulated genes in the EA.hy926 cells after exposure to SP, SOA_βPIN_-SP or SOA_NAP_-SP compared to the CA control are summarized in Tables S5,S6 and S7, respectively. An overlap of approximately 41% of similar DEGs after all three aerosol exposures was observed in EA.hy926 cells. The most uniquely expressed genes were detected after exposure to SOA_NAP_-SP (25%), followed by SOA_βPIN_-SP (8%) and SP (7%) (Fig. 2E). For the significantly upregulated genes in EA.hy926 cells, GO analysis revealed enrichment of GO terms, such as “receptor activity”, “transcription activity”, “angiogenesis”, and “inflammation” for all aerosol types; however, there was a greater –log_10_(p-value) for SOA_NAP_-SP (Fig. 2F). These findings suggested an early involvement of endothelial cell biology in systemic effects and are consistent with the findings of a study in healthy individuals highlighting endothelial cell injury and systemic inflammation caused by PM_2.5_ exposure [6].

### Aerosol exposure to the tested aerosols induced stress- and inflammation-related gene expression in both epithelial (A549) and endothelial (EA.hy926) cells

**Fig. 3:**
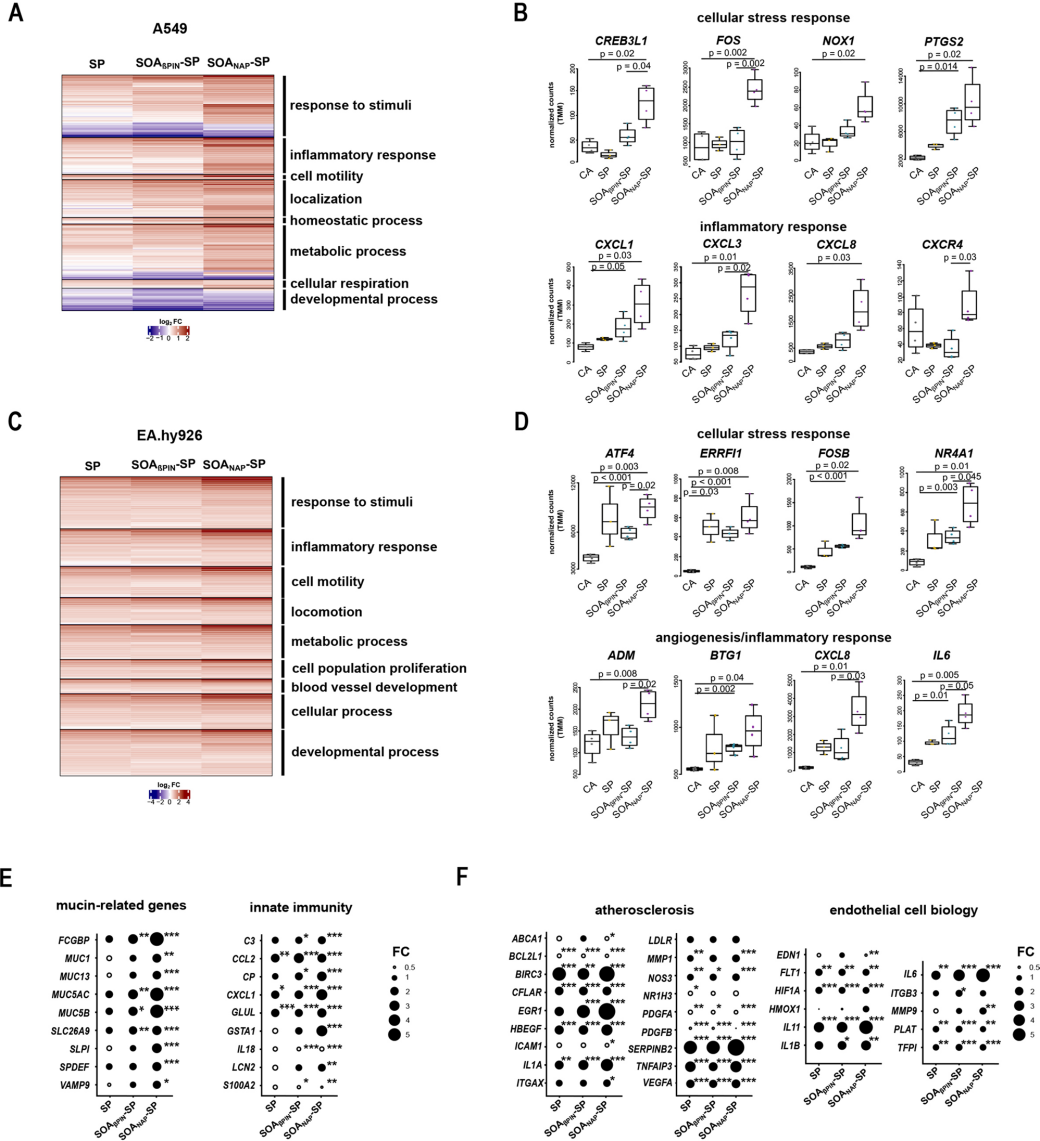
Induction of stress- and inflammatory-related genes in A549 and EA.hy926 cells. (**A**) Heatmap comparing gene expression values among different aerosol exposures (SP, SOA_βPIN_-SP and SOA_NAP_-SP) compared to those of the clean air (CA) control originating from GO overrepresentative analysis of A549 cells. (**B**) Boxplots of the genes depicted from the heatmap in Fig. 3A showing the normalized counts (TMM) in A549 cells after exposure to CA, SP, SOA_βPIN_-SP or SOA_NAP_-SP. (**C** – **D**) Same as in (**A** and **B**) but based on the analysis of EA.hy926 cells. (**E** and **F**) Dot plots showing the level of expression (fold change) of genes activated (filled dots) or suppressed (empty dots) by exposure to SP, SOA_βPIN_-SP or SOA_NAP_-SP compared to CA exposure in the “mucin-related genes” and “innate immunity” categories for A549 cells I and in the “atherosclerosis” and “endothelial cell biology” categoriesfor EA.hy926 cells (**F**). * adjusted p-value < 0.05, ** adjusted p-value < 0.01 and *** adjusted p-value < 0.001

To expand our understanding of the cellular effects to different SOA types, we next characterized the aerosol-specific impact on biological processes. We first performed a gene set enrichment analysis (GSEA) on all the genes, which detected small but consistent changes in a predefined set of genes. We sorted the resulting terms by a normalized enrichment score (NES) of either ≥ 2 or ≤ -2, categorized the remaining terms into their respective GO ‘parent’ terms (Excel Sheets S1 and S2) and clustered the comprised significant DEGs in a heatmap. In A549 cells, exposure to SOA_NAP_-SP induced the upregulation of genes associated with the enriched terms for “response to stimuli”, “inflammatory response”, and “cell motility” (Fig. 3A and Excel Sheet S1). Moreover, we found more highly expressed cellular stress response genes, such as cAMP responsive element binding protein 1 (*CREBL1*) and fos proto-oncogene (*FOS*) (Fig. 3B), which are known to be involved in early endoplasmic reticulum (ER) stress and oxidative stress. This effect was less detectable after exposure to SP or SOA_βPIN_-SP. Several studies have linked cellular ER stress [32] and reactive oxygen species (ROS) generation [33] to redox-active quinones. Given that SOA_NAP_-SP contains more quinones, such as 1,2- and 1,4-naphthoquinone, we hypothesized that these quinones would impact on gene expression. Moreover, the significantly enhanced expression of the gene NADPH oxidase 1 (*NOX1*) (Fig. 3B) possibly indicates the occurrence of ROS [34], which is consistent with the observed elevated levels of the oxidative stress marker malondialdehyde (MDA) after exposure to SOA_NAP_-SP compared to those after exposure to SP and SOA_βPIN_-SP [21]. The increased expression of the gene prostaglandin-endoperoxidase synthase 2 (*PTGS2*) is likely due to its protective function against pulmonary oxidative stress [35] and acute lung injury [36]. This finding is in line with a study showing enhanced cellular expression of *PTGS2* after exposure to diesel exhaust (PM_2.5_) [37]. In addition to demonstrating an increase in stress-related gene expression, we also observed the induction of inflammatory response genes [e.g., CXC motif chemokine ligand 1, 3 and 8 (*CXCL1*, *CXCL3* and *CXCL8*) and CXC motif chemokine receptor 4 (*CXCR4*)] (Fig. 3B) after exposure to SOA_NAP_-SP, which are among the other powerful neutrophil chemoattractants and are involved in the pathogenesis of airway inflammation, asthma, cancer and angiogenesis [38–40]. Similarly, EA.hy926 cells demonstrated strong enrichment of genes within the categories, e.g., response to stimuli, inflammatory responses, blood vessel development and cell population proliferation (Fig. 3C and Excel Sheet S2). Consistent with the findings in A549 cells and consistently with the observation of secondary genotoxicity in EA.hy926 cells by comet assay in a previous study [21], we found significantly upregulated oxidative stress and ER stress-related genes, such as activating transcription factor-4 (*ATF4*) (Fig. 3D). *ATF4* serves as the master transcriptional regulator of the cellular response to ER stress or amino acid starvation, controls genes involved in metabolism and protection from oxidative stress [41] and apoptosis or senescence [42] and can be linked to angiogenesis [43] and metastasis [44]. Moreover, the activation of ErbB receptor feedback inhibitor 1 (*ERRFI1*), the FosB proto-oncogene *(FOSB*), and nuclear receptor subfamily 4 group a member 1 (*NR4A1*), has been associated with immediate early genes, especially after exposure to SOA_NAP_-SP (Fig. 3D). Early genes exhibit rapid transcription in response to acute stress, such as hypoxia or hyperglycemia [45, 46], or proliferation-inducing signals possibly accompanied by genomic instability and activated DNA damage responses [47]. In addition to the activation of downstream genes related to early stress responses, genes involved in the inflammatory response are also often targeted. Here, we detected significant enrichment of adrenomedullin (*ADM*), B-cell translocation gene 1 (*BTG1*), *CXCL8* and interleukin 6 (*IL6*) after exposure to SOA_NAP_-SP and, to a lesser extent, to SOA_βPIN_-SP (Fig. 3D). *ADM* is activated in the presence of proinflammatory cytokines (e.g., IFNγ, IL1β, and IL6), that contributes to endothelial and epithelial barrier functions [48], and impacts the blood and lymphatic vasculature [49]. In endothelial cells, overexpression of *BTG1* promotes cell migration and tube formation [50], upregulation of *CXCL8* and *IL6* can be associated with cellular dysfunctions [51], and elevated serum levels serve as markers for adverse prognosis [52, 53]. Several studies have shown a correlation between endothelial inflammatory activation and endothelial dysfunction and the development of atherosclerosis or hypertension [54, 55].

Intriguingly, by examining the most up- or downregulated genes (Table S3), we detected high expression of mucin-related genes, such as fc gamma binding protein (*FCGBP*), mucin 1 (*MUC1*) and mucin 5AC (*MUC5AC*) (Fig. 3E), which have been previously associated with stress-related airway remodeling processes, in the epithelial cells after SOA_NAP_-SP exposure [56, 57]. Moreover, we found differential expression of more mucin-related genes, such as mucin 13 (*MUC13*), mucin 5B (*MUC5B*), solute carrier family 26 (*SLC26A9*), *SLIP*, SAM pointed domain containing ETS transcription factor (*SPDEF*) and vesicle-associated membrane protein 9 (*VAMP9*). In addition, SP exposure also led to the induction of innate immune-specific genes in A549 cells; however, this effect was lower than that of SOA_βPIN_-SP and SOA_NAP_-SP exposure (Fig. 3E). Collectively, these findings possibly point toward induced stress-related airway remodeling in AT2-secretory like A549 cells to more MUC5A^+^ goblet-secretory-like cells and an early type I immune response after exposure to SOA_NAP_-SP. In 2005, Kunzli, Jerrett [58] published the first epidemiological study showing a correlation between atherosclerosis and ambient air pollution (PM_2.5_). In our model system, endothelial cells also responded by upregulating several atherosclerosis marker genes after all aerosol exposures (Fig. 3F). Marked upregulation of the genes baculoviral IAP repeat containing 3 (*BIRC3*), epidermal growth factor receptor (*EGFR3*), interleukin 1 alpha (*IL1A*) and serpin family b member 2 (*SERPINB2*) was detected after exposure to SOA_NAP_-SP (Fig. 3F). When evaluating more known endothelial cell biology genes, we further found altered genes involved in angiogenesis (e.g., fms-related receptor tyrosine kinase 1; *FLT1*), vasodilation and constriction (e.g., endothelin 1; *EDN1*), coagulation (e.g., plasminogen activator; *PLAT*, tissue factor pathway inhibitor; *TFPI*) and platelet activation (e.g., interleukin 11; *IL11*, interleukin 6; *IL6*) (Fig. 3F). These findings revealed a strong increase in proatherosclerotic, procoagulation and platelet-activated gene expression. Taken together, our results suggested early activation of stress-related and pathogenic-specific genes in the direct exposed A549 epithelial cells, but also in the beneath laying EA.hy926 endothelial cells, highlighting the importance of using multicellular coculture systems for exposure studies.

### Circulating cytokine validated the observed toxicogenomic responses

**Fig. 4:**
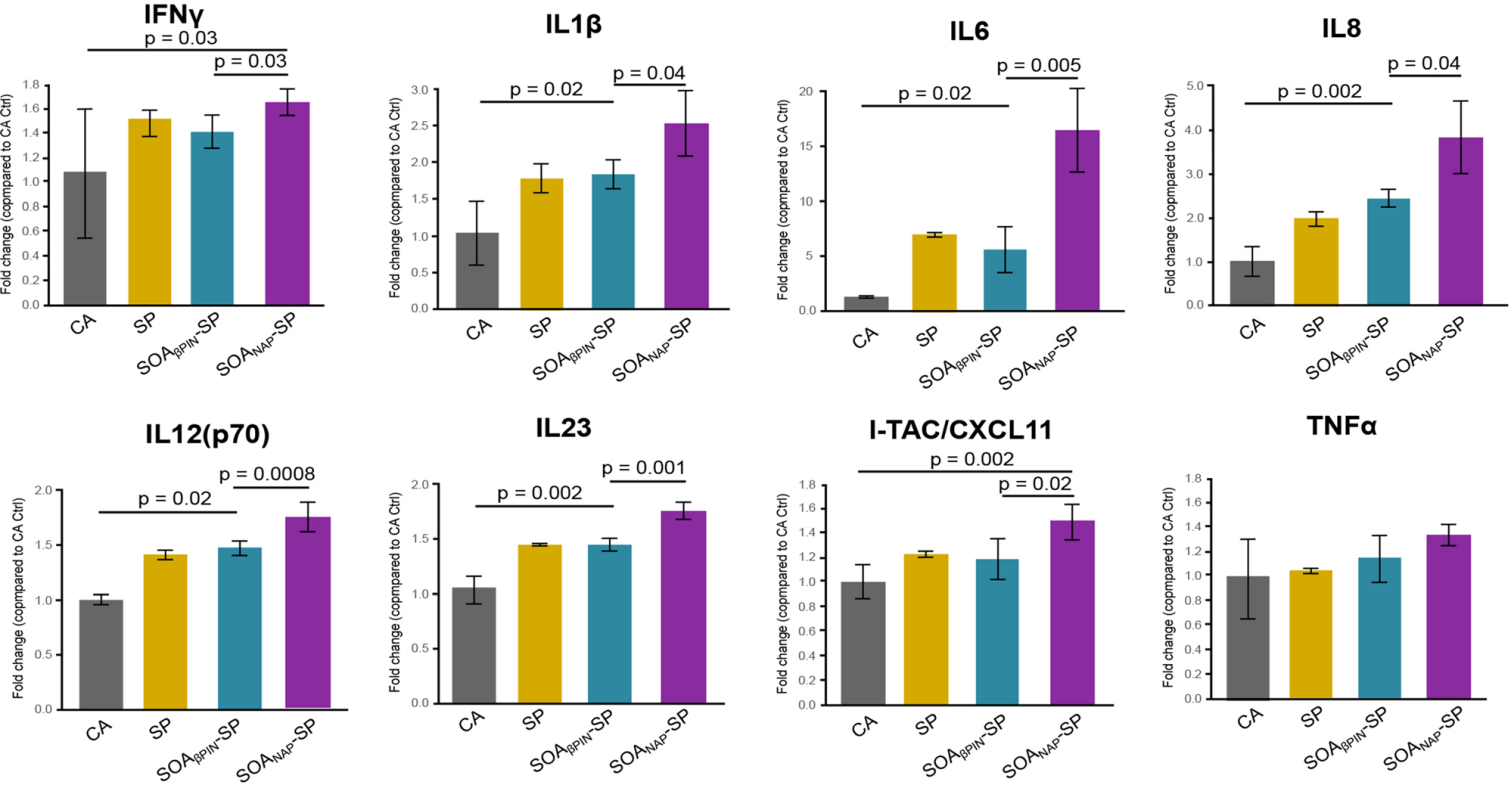
Circulating cytokine validated the observed toxicogenomic responses. Cytokine validation of IFN-γ, IL1-β, IL6, IL8, IL12(p70), IL23, I-TAC/CXCL11 and TNFα in the cell culture media of the coculture system after exposure to CA, SP, SOA_βPIN_-SP and SOA_NAP_-SP. The data are presented as the fold change (compared to the CA control) ± SEM from three independent experiments, and the significance of the differences are outlined

Transcriptional changes were validated by analyzing the conditioned cell culture media for cytokines. These cytokines were chosen based on the RNAseq results and subsequently correlated with PM-induced cell responses [20, 59]. The observed cytokine secretion and transcription profiles were concordant with cytokines IL-1β, IL6, IL8 and IL12, which were found among the top upregulated genes in A549 and EA.hy926 cells after exposure to SOA_NAP_-SP. These cytokines can induce the activation of several observed upregulated genes (*CXCR4* and *ADM*), as described above (Fig. 4, Tables S5 and S8). Moreover, significant differences in cytokine secretion were detected for IL12(p70) and IL23 after SOA_βPIN_-SP exposure and for IFNγ, IL12(p70), IL23 and CXCL11 after SOA_NAP_-SP exposure. No significant change was observed for TNFα (Fig. 4). It is suggested that the secretion of IL1β, IL6 and IL8 by epithelial cells is coupled to the organic compounds in the particle phase, as previously shown for diesel engine exhaust PM [60] and ambient PM_2.5_ [61]. This possibly explains the minor secretion of cytokines by epithelial cells exposed to uncoated pure SP. Additionally, the activation of the aryl hydrocarbon receptor (AHR) by polycyclic aromatic hydrocarbons (PAHs), such as naphthalene or its ring-retaining photooxidation products, was shown to be responsible for inflammatory responses via the upregulation of IL6 through nuclear factor kappa-B NFκB signaling [62] and the induction of lung cancer in mice [63]. Here, we observed significantly increased expression of the *AHR* and *NFκB2* genes after exposure to SOA_NAP_-SP in A549 cells (*AhR*: log_2_FC: 0.93, *p* < 0.001; *NFkB2*: log_2_FC: 0.51, *p* = 0.012); however, at our tested time points we were not able to detect any upregulation of the cytochrome P450 genes, which are known to play an important role in the metabolism of early-generation aging products of naphthalene, e.g., 1-naphthol and 1,2-dihydroxynaphthalene [64]. Moreover, we detected enrichment of cytokines from the IL12 family (IL12 and IL23) and CXCL11 in the cell culture media, especially after exposure to SOA_NAP_-SP, which plays a critical role in the development of T-cell-mediated immunity [65], and elevated levels of these cytokines appeared in patients with asthma, acute respiratory diseases and autoimmunity [66–68].

### Integrative analysis of circulating metabolites and gene expression revealed important role of non-directly exposed endothelial cells

**Fig. 5:**
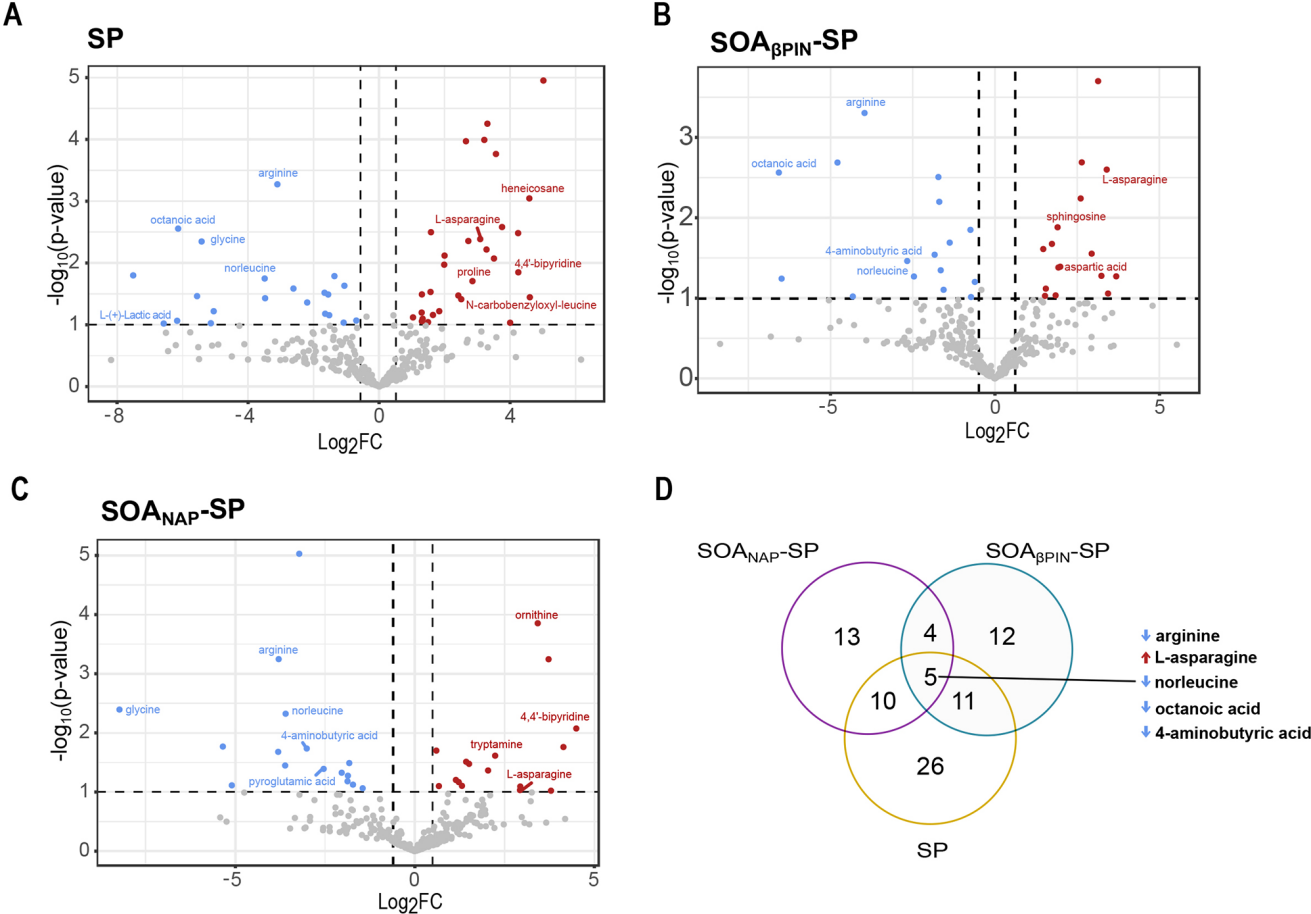
Integrative analysis of circulating metabolites and gene expression revealed important role of non-directly exposed endothelial cells. (**A**-**C**) Volcano plots showing the relationships between differentially abundant metabolite expression (p-value ≤ 0.1 and log_2_FC ≥ 0.5 and log_2_FC ≤ -0.5) in the cell culture media after exposure to SP (**A**), SOA_βPIN_-SP (**B**) or SOA_NAP_-SP (**C**) and the CA control. (**D**) Venn diagram of the unique and overlapping metabolites identified as up- or downregulated (p-value ≤ 0.1, log_2_FC ≥ 0.5 and log_2_FC ≤ -0.5) in the cell culture media after exposure to SP, SOA_βPIN_-SP or SOA_NAP_-SP compared to the CA control. GC/MS analysis was performed on *n* = 3 independent experiments

In recent years, invaluable information has been obtained from metabolomics to mechanistically understand the impact of PM_2.5_ exposure. Thus, we next investigated the metabolic signature defined by exposure to SP, SOA_βPIN_-SP or SOA_NAP_-SP in the collected sample media. We identified a total of 312 metabolites, with 51 differentially expressed metabolites after SP exposure (Fig. 5A and Excel Sheet S3), 32 after SOA_βPIN_-SP exposure (Fig. 5B and Excel Sheet S3) and 32 after SOA_NAP_-SP exposure (Fig. 5C and Excel Sheet S3), with respect to the CA control. Here, we observed distinct metabolite profiles, with only five metabolites commonly found to a greater or lesser extent in the exposure media after exposure to both SOA types and SP and with metabolites allocated to cellular and potential aerosol origins (Fig. 5D). In particular, the accumulation of chemical compounds of non-endogenous origin, such as i.e., 1,2,3-cyclohexanetriol after the exposure to SOA_βPIN_-SP or 3,4,5-trihydroxybenzyl methyl ether and 3-hydroxy-2-naphthoic acid after the exposure to SOA_NAP_-SP can possibly be correlated with oxidation compounds of the respective aerosols. Interestingly, in the toxicogenomic database [69], 3-hydroxy-2-naphthoic acid has been associated with an interaction of G-protein-coupled receptor 35 (GPR35), whose increased activity has been linked to various pathologies in the inflammatory and cardiovascular systems [70] or to a poor prognosis in non-small lung cancer [71].

Like a study by Breitner, Schneider [72] investigating the abundance of metabolites in blood plasma after short-term exposure to PM_2.5_ in a cardiac catheterization cohort, we detected a decrease in arginine and glycine. In contrast, ornithine was significantly enriched after exposure to SOA_NAP_-SP. This finding was consistent with the results for glycine degradation (SOA_NAP_-SP, *p* < 0.001; SOA_βPIN_-SP, *p* = 0.048 and SP, *p* = 0.013), the urea cycle (SOA_NAP_-SP, *p* = 0.006 and SOA_βPIN_-SP, *p* = 0.01) and arginine degradation (SOA_NAP_-SP, *p* < 0.006; and SP, *p* = 0.015) under the 5 most altered canonical pathways identified by IPA analysis (Table 2). An increased consumption of glycine by cells was observed to be a sign of glutathione (GSH) demand induced through oxidative stress [73] or angiogenesis through vascular endothelial growth factor (VEGF) signaling [74]. Moreover, a reduced serum level of glycine in patients with COPD has been correlated with airflow obstruction via the development of emphysema [75].

**Table 2:** Canonical pathway enrichment analysis of the metabolome data

Canonical Pathways	SOA_NAP_-SP	SOA_βPIN_-SP	SP
Glycine Degradation (Creatine Biosynthesis)	**< 0.001**	**0.048**	**0.013**
tRNA Charging	**0.029**	**0.006**	**0.002**
Macrophage Alternative Activation Signaling Pathway	**0.006**	**0.048**	**0.013**
Urea Cycle	**0.006**	**0.010**	0.075
Arginine Degradation VI (Arginase 2 Pathway)	**0.006**	0.051	**0.015**
Proline Biosynthesis II (from Arginine)	**0.006**	0.056	**0.017**
Arginine Biosynthesis IV	**0.008**	**0.014**	0.089
Superpathway of Citrulline Metabolism	**0.013**	**0.022**	**0.045**
Citrulline Biosynthesis	**0.008**	0.070	**0.029**
Arginine Degradation I (Arginase Pathway)	**0.006**	0.051	0.070
Nitric Oxide Signaling in the Cardiovascular System	**0.006**	0.056	0.073
Citrulline-Nitric Oxide Cycle	**0.049**	**0.008**	0.073
Glutamate Dependent Acid Resistance	**0.028**	**0.046**	0.066
γ-glutamyl Cycle	**0.006**	-	**0.017**

*Note* The canonical pathways associated with the genes whose expression significantly changed according to the metabolome analysis of the cell culture media exposed to SOA_NAP_-SP, SOA_βPIN_-SP or SP are shown. The results are shown as BH-corrected p-values from three independent experiments (*n* = 3). Significant values are shown in bold (*p* ≤ 0.05)

In addition, the reduced availability of arginine and increased abundance of ornithine in the cell culture media suggested enhanced activity of the enzyme arginase within the urea cycle. This has already been observed in conditions of acute and chronic stress [76] and in a study by Liang, Ladva [77] in which asthmatic persons were exposed to traffic-related pollutants. Interestingly, reduced secretion of 4-aminobutyric acid (GABA) was detected after all aerosol exposures, and increased uptake of GABA in airway epithelial progenitor cells induced differentiation into goblet cells [78], supporting our RNASeq analysis. As anticipated, we detected alterations in gene and metabolite expression; however, the degree to which transcriptomic and metabolic changes occur has remained elusive. Therefore, to integrate the metabolome data with the gene expression data, we performed an IPA comparison analysis with a pathway-based approach [79]. We found that eight common pathways were significantly altered in the gene expression of A549 and/or EA.hy926 cells and in the metabolome of the sampled media after all aerosol exposures (Table 3). The iNOS signaling pathway was altered in EA.hy926 cells after all exposures at the transcript level (SOA_NAP_-SP: *p* = 0.013; SOA_βPIN_-SP: *p* = 0.007 and SP: *p* = 0.019) and at the metabolome level after exposure to SOA_NAP_-SP (*p* = 0.037) as well as SOA_βPIN_-SP (*p* = 0.048). The PDGF signaling pathway was significantly regulated in both cell lines following exposure to SOA_NAP_-SP (A549: *p* = 0.029; EA.hy926: *p* = 0.004) and in only EA.hy926 cells after exposure to SOA_βPIN_-SP (*p* = 0.039) or SP (*p* = 0.027). However, at the metabolome level, the PDGF pathway was regulated only after exposure to SOA_βPIN_-SP (*p* = 0.048). These pathways have previously been shown to be involved in vascular endothelial cell migration [80] and the development of fibrotic diseases [81]. Notably, after exposure to SOA_NAP_-SP, we found an alteration in phosphatidylinositol 3-kinase (PI3K)/protein kinase B (AKT) signaling in the gene expression of EA.hy926 cells in response to SOA_NAP_-SP (*p* = 0.016) and in the metabolome data after exposure to both SOA_NAP_-SP (*p* = 0.042) and SOA_βPIN_-SP (*p* = 0.048). This indicated the involvement of DNA damage and oxidative stress [82] as well as the typical activation of endothelial cells for effective angiogenesis [83], as demonstrated at the functional level by the observed secondary genotoxicity and induced angiogenesis [21]. Taken together, these findings indicate that exposure to SP, SOA_βPIN_-SP and SOA_NAP_-SP is associated with significant metabolomic changes that are distinct for single metabolites, leading to altered phenotypes and highlighting the pivotal role of not directly exposing EA.hy926 cells.

**Table 3:** Canonical pathway enrichment analysis of the transcriptome and metabolome data via IPA software

Canonical Pathways	A549			EA.hy926			Metabolome		
	SOA_NAP_-SP	SOA_βPIN_-SP	SP	SOA_NAP_-SP	SOA_βPIN_-SP	SP	SOA_NAP_-SP	SOA_βPIN_-SP	SP
iNOS Signaling	0.436	-	-	**0.013**	**0.007**	**0.019**	**0.037**	**0.048**	0.067
PDGF Signaling	**0.029**	0.674	0.520	**0.004**	**0.039**	**0.027**	-	**0.048**	0.067
Corticotropin Releasing Hormone Signaling	0.269	0.867	0.665	**0.037**	0.130	**0.017**	**0.046**	0.051	0.070
PI3K/AKT Signaling	0.577	-	0.832	**0.016**	0.060	0.097	**0.042**	**0.048**	0.067
Ceramide Signaling	0.484	-	-	**0.006**	**0.050**	0.067	-	**0.048**	0.067
Apelin Endothelial Signaling Pathway	0.469	0.793	0.790	**0.013**	0.538	0.109	**0.042**	**0.048**	0.067
Sphingosine-1-phosphate Signaling	0.225	0.671	0.679	**0.004**	0.152	0.193	-	**0.048**	0.067
Inhibition of Angiogenesis by TSP1	0.619	0.904	-	**0.028**	0.619	0.544	**0.042**	**0.048**	0.067

*Note* The canonical pathways associated with the significantly altered A549 and/or EA.hy926 transcript levels are shown, as is the metabolome analysis of the cell culture media exposed to SOA_NAP_-SP, SOA_βPIN_-SP or SP. The results are shown as BH-corrected p-values from four (RNASeq; *n* = 4) and three (metabolome; *n* = 3) independent experiments. Significant values are shown in bold (*p* ≤ 0.05)

## Limitations of the study

Despite the importance of these findings, there are limitations to our study that should be considered. First, by choosing A549 cells as our epithelial lung cell line we are aware of several possible drawbacks, including a low expression of tight junctions and adherence proteins, inability to form a fully polarized epithelium, as well as, being of carcinogenic origin [84]. Therefore, additional studies are needed to observe the effects of SOA exposure on other epithelial cell lines or even primary human epithelial cells. However, due to the complexity of our experimental setup, we found with A549 cells a robust and metabolic competent cell line that is suitable for large and repetitive studies. Second, the focus on only one aerosol dilution (1:3) and one time point (4 h exposure) complicates the risk assessment of the aerosols. Future studies comparing short- and long-term exposures and different aerosol doses should help characterize meaningful adverse effects.

## Conclusion

**Fig. 6:**
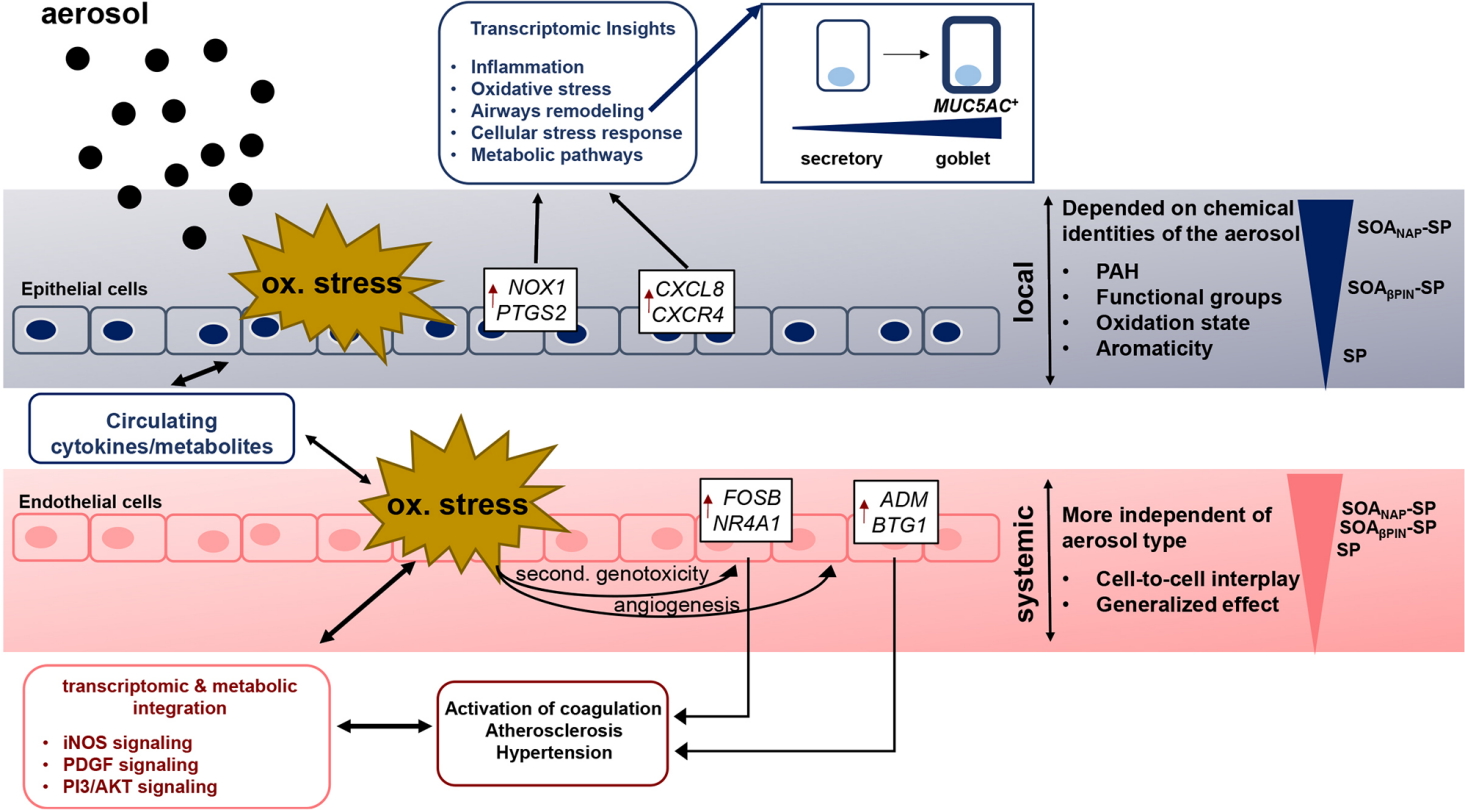
Proposed pathomechanisms triggered by aerosol exposure. Aerosols confer most of their effects via damage to the lung (inflammation, oxidative stress, genotoxicity, and airway remodeling toward MUC5AC^+^ goblet-secretory like cells). This effect seemed to be dependent on the chemical composition of the specific aerosols (organic content, PAHs, functional groups, oxidation state, aromaticity) and was greatest after exposure to SOA_NAP_-SP, followed by SOA_βPIN_-SP and SP. The primary target organ damaged by aerosols converges at the cardiovascular level by inducing dysfunctions in endothelial cells (secondary genotoxicity), inflammation, metabolic reprogramming, and angiogenesis. These effects seemed to be more independent of the aerosol type and systemic treatment, resulting in the activation of coagulation, atherosclerosis and/or hypertension via iNOS, PDGF and/or PI3/AKT signaling, respectively

In this study, we have revealed the importance of the chemical identities of SOA in inducing different toxicogenomic and metabolic effects in an epithelial-endothelial coculture system (Fig. 6). By conducting exposure studies with physically similar but chemically distinct model SOA, we identified aerosol-specific effects depending on chemical composition, especially in directly exposed epithelial cells (A549). The exposure to SOA derived from an anthropogenic aromatic precursor (naphthalene) resulted in the upregulation of genes in A549 cells related to oxidative stress (e.g., *NOX1* and *PTGS2*) and to an early type I immune response (e.g., *CXCL8* and *CXCR4*) in A549. In addition, transcriptomic insights revealed a stress-related airway remodeling of AT2-secretory like cells to more MUC5A^+^ goblet-secretory-like cells. Milder effects were observed in A549 cells exposed to a biogenic, aliphatic SOA precursor (β-pinene) and fresh SP. Remarkably, we detected early activation of stress- (e.g., *FOSB* and *NR4A1*) and CVD-related genes (e.g., *ADM* and *BTG1*) in the underlying endothelial cells (EA.hy926). This effect was significant after exposure to SOA_NAP_-SP and minor after exposure to SOA_βPIN_-SP or SP and in line with the previously detected DNA breaks and increased angiogenic potential. Cytokine and metabolic analyses confirmed the induction of a systemic proinflammatory state, and the integration analysis highlighted the key role of cells that are not directly exposed in the overall molecular toxicological response. We observed alterations especially in signaling pathways that are known to play key roles in the onset of CVD (e.g., iNOS, PDGF and PI3/AKT signaling). Our findings thus emphasize the importance of enabling cellular crosstalk in exposure assessment studies combined with systems biology approaches to better determine potential human health risks beyond the lung due to exposure to SOA.

## Materials and methods

### Aerosol generation and PM (SOA) characterization

A detailed description of the aerosol generation and characterization has been given previously [20, 21, 85]. Briefly, secondary organic aerosols (SOA) of either naphthalene (Sigma-Aldrich, 147141-25G, 99%) or β-pinene (Sigma-Aldrich, 402753-10G, ≥ 99%) were produced by mixing their pure vapor with soot particles (SP) from a Combustion Aerosol Standard generator (CAST, model 5201 C; Jing Ltd., Zollikofen, CH). The mixtures were subsequently processed in an oxidation flow reactor (potential aerosol mass reactor, PAM, Aerodyne Research Inc., Billerica, MA, US) [86, 87] for the simulation of atmospheric photooxidation (aging) dominated by OH radicals. This process resulted in two SOA types, SOA_βPIN_-SP and SOA_NAP_-SP. Pure SP were fed into the PAM and used as condensation nuclei in the SOA experiments, without precursor addition or aging, as a reference. Methanolic extracts of SOA-SP were examined by ultrahigh-resolution Fourier-transform ion cyclotron resonance mass spectrometry (SolariX, 7 T FT-ICR MS, Bruker Daltoniks, Bremen, GE) with electrospray ionization in positive and negative mode with detailed parameters discussed previously elsewhere [20] to determine the elemental composition. The elemental composition was determined by the exact mass identified at a signal-to-noise ratio (S/N) of 9. The following settings were applied for elemental composition assignment: C_c_H_h_O_o_Na_x_; for positive mode ESI: 2 ≤ c ≤ 100, 2 ≤ h ≤ 100, o ≤ 20, and x ≤ 1; and for negative mode ESI: 2 ≤ c ≤ 50, 2 ≤ h ≤ 100, o ≤ 16, and x ≤ 0 with a maximum error of 2 ppm. To describe the degree of unsaturation, the concept of double bond equivalents (DBE) is used, considering that the octet rule (except for hydrogen) is obeyed and that unsaturation is caused by covalent carbon – carbon bonds.

1 $$DBE = 1 + C - 0.5H + 0.5N$$

The MCR is calculated from the relation of DBE to the number of oxygen atoms (#O) via the sum formula.

2 $$MCR = {{DBE} \over {\# O}}$$

### Gas phase characterization

VOC in the gas phase were analysed and quantified via proton-transfer-reaction (PTR) high-resolution time-of-flight mass spectrometry (PTR-TOFMS; IONICON Analytik GmbH, Innsbruck, AT) via measurements of the filtered gas phase at the outlet of the PAM at a 1:10 dilution using previously described settings and procedure in [88].

### Cell culture and ALI exposure

Detailed descriptions of the cell culture and the ALI exposure systems used are given in Offer, Hartner [21]. Briefly, A549 human alveolar epithelial cells (ATCC^®^, CCL-185™) and EA.hy926 human endothelial cells were routinely cultured in high-glucose Gibco Dulbecco’s Modified Eagle Medium: Nutrient Mixture F-12 (DMEM/F-12) (ThermoFisher Scientific, 31331-028) supplemented with 5% (v/v) fetal bovine serum (FBS) (ThermoFisher Scientific, 10500-064), 100 U/mL penicillin, and 100 µg/mL streptomycin (P/S; Sigma-Aldrich, P4333) in a humidified incubator at 37 °C and 5% CO_2_. For the coculture exposure experiments, A549 cells were seeded on transferrable 24 mm Transwell^®^ inserts with a polyester membrane (0.4 μm pore-size, Type #3450, Corning, NY, US) 96 h before the exposure experiments at a density of 1.8 × 10^5^ cells/mL per insert (3.8 × 10^4^ cells/cm^2^ growth area) with 1.5 mL of cell culture medium provided at the basal compartment and 1 mL at the apical side of the Transwell^®^ plate. Forty-eight hours after the initial cell seeding, the culture medium on the apical side was removed to establish ALI conditions, and fresh cell culture medium (1.5 mL) was added to the basal compartment. After an additional 24 h later, the inserts were inverted, 1 × 10^5^ EA.hy926 cells per insert (0.21 × 10^4^ cells/cm^2^ growth area) were seeded in 750 µl of medium, and after 1 h, the insert was returned, after which fresh medium (1.5 mL) was added to the basolateral compartment of the Transwell^®^ plate. On day 5, the day of the exposure experiments, the inserts were placed in the exposure modules of the ALI exposure system (Vitrocell^®^ Automated Exposure Station Standard Version, Vitrocell Systems, Waldkirch, GE) with 1.8 mL of serum-free DMEM/F12 medium supplemented with 1% P/S and 15 mM N-2-hydroxyethylpiperazine-N-2-ethane sulfonic acid (HEPES) buffer solution (ThermoFisher Scientific, 15630-056) in the basolateral compartment. The cells were then exposed for 4 h to conditioned (85% r.h. 37 °C) and 1:3 diluted aerosol (PM in the gas phase), named SP, SOA_βPIN_-SP and SOA_NAP_-SP with a 100 mL/min flowrate over every position in the ALI exposure system, as described in Mülhopt, Dilger [89]. Due to trumpet-shaped flow-guiding elements in every position of the ALI exposure system, the aerosols are depositing onto cells by diffusion and sedimentation [90]. In addition to the aerosol exposures, each system had a separate clean air (CA; purified compressed laboratory air) exposure sector serving as a control. Aerosol exposure to SP was conducted in *n* = 3 independent experiments, to SOA_βPIN_-SP in *n* = 4 independent experiments and to SOA_NAP_-SP in *n* = 4 independent experiments. To avoid possible sources of inherent variability in the deposited aerosols due to the ALI exposure system, the same insert positions were always used per module. After exposure, the effects of the aerosols and CA on cells were examined via several assays (RNASeq analysis), and the exposure medium was collected (from the sample media) on ice and frozen at -80 °C for later analyses (cytokine and metabolome analysis).

### RNA extraction, library construction and sequencing

Transwell^®^ inserts were placed in a 6-well plate (ThermoScientific, 140675) containing RNAprotect Cell Reagent (QIAGEN, Hilden, 76526) immediately after exposure. Then, the A549 cells on the apical side and the EA.hy926 cells on the basolateral side were carefully scraped with a cell scraper (Sigma, SIAl0010) and separately stored at -20 °C until RNA extraction. Total RNA was extracted from the cells using an RNA Plus Mini Kit (QIAGEN, Hilden, 74136) according to the manufacturer’s instructions. The quality of the extracted RNA was assessed with a Nanodrop and TapeStation (Agilent Technologies, CA, US) and only replicates with high RNA integrity (RIN > 9.8) were processed for RNA sequencing (RNASeq) at the Crown Genomics Institute of the Nancy and Stephen Grand Israel National Center for Personalized Medicine, Weizmann Institute of Science. Total RNA (500 ng for each sample) was processed using the inhouse poly A-based RNAseq protocol (INCPM mRNA Seq). SR reads were sequenced on 1 lane(s) of an Illumina NovaSeq Sp (100 cycles) with an output of ~ 17 million reads per sample. Libraries were evaluated by Qubit (Thermo FisherScientific Inc., MA, US) and TapeStation (Agilent Technologies, CA, US) instruments. A detailed description of the RNA extraction, library construction and sequencing procedure was previously described in [20]. Shortly, P Poly-A/T stretches and Illumina adapters were trimmed from the reads using cutadapt [91], resulting reads shorter than 30 bp were discarded. Reads were mapped to the H. sapiens reference genome GRCh38 using STAR [92], supplied with gene annotations downloaded from Ensembl (and with EndToEnd option and outFilterMismatchNoverLmax was set to 0.04). The percentage of the reads aligned uniquely to the genome was 97%. Reads with the same UMI were removed using the PICARD MarkDuplicate tool using the BARCODE_TAG parameter. Expression levels for each gene were quantified using htseq-count (version 0.11.2) [93], using the gtf above. Only uniquely mapped reads were used to determine the number of reads falling into each gene (intersection-strict mode). The sequencing data have been deposited in the NCBI´s Gene Expression Omnibus [94] and are accessible through the GEO Series accession number GSE226350 (https://www.ncbi.nlm.nih.gov/geo/query/acc.cgi?acc=GSE226350).

### Differential gene expression analysis

All the statistical analyses were performed in the R programming language (version 4.1.2). For the A549 and EA.hy926 samples, genes with a count per million (CPM) reads ≥ 30 in at least 3 samples (the number of the fewest replicates of a given condition) were retained. Gene expression levels were normalized by the trimmed mean of M-values (TMM) method using the calcNormFactors function of the EdgeR package in R [95]. Moreover, samples were corrected based on the processed sequencing batch, and differential expression analyses were performed using the limma-voom package in R [96]. Significantly differentially expressed genes (DEGs) of all aerosol types compared to those of the clean air (CA) control were selected with a controlled FALSE positive rate (BH method) of 5% (FDR ≤ 0.05). Upregulated genes were selected at a minimum log_2_-fold change of 0.5, and downregulated genes were selected at a minimum log_2_-fold change of -0.5. Principal component analyses (PCA) were performed using batch effect-corrected normalized values of the genes expressed by A549 and EA.hy926 cells. Heatmaps were drawn on the normalized expression matrix using the heatmap.2 function from the gplots package in R with Euclidean distance and complete linkage for hierarchical clustering [97]. To visualize the statistical significance versus the log_2_-fold change, volcano plots were generated by using the EnhancedVolcano plot function in R [98]. Venn diagrams were constructed using the vennDiagram function based on the overlapping DEGs (FDR ≤ 0.05, log_2_-fold change ≤ -0.5/ ≥ 0.5).

### Gene Ontology (GO) overrepresentation analysis and GO gene set enrichment analysis

Gene Ontology (GO) overrepresentation analysis of the biological process (BP) and molecular function (MF) categories was performed using the enrichGO function from the clusterProfile package in R [99] based on DEGs (FDR ≤ 0.05, log_2_-fold change ≥ 0.5) of A549 and EA.hy926 cells, respectively. Important GO terms were selected based on an FDR ≤ 0.05, and the top ten results were plotted by bar plots showing the –log_10_(p-value) of the analysis. Gene set enrichment analysis (GSEA) was performed by the gseaGO function from the clusterProfile package in R, which computes the normalized enrichment score (NES) for each cell line and condition. For our analysis, only pathways with a NES above 2 or below − 2 were selected and further analysed by categorization into their respective GO ‘parent’ terms [100, 101], which was followed by clustering of the significant DEGs (FDR ≤ 0.05, log_2_-fold change ≥ 0.5) in a heatmap generated with the heatmap function of the complexHeatmap package in R [102].

### Plotting specific gene expression across aerosol exposure

Using heatmaps, we identified genes within the functional GO gene sets (“response to stimuli” and “inflammatory response”) and created boxplots on the basis of the normalized counts (TMM). Moreover, categories were generated with the names “mucin-related gene” and “innate immunity”, in which we plotted the fold change in DEGs in A549 cells (FDR ≤ 0.05, log_2_-fold change ≥ 0.5 or ≤ -0.5) that have been previously published within those contexts [56]. For EA.hy926 cells, we focused on genes (FDR ≤ 0.05, log_2_-fold change ≥ 0.5 or ≤ -0.5) that have been used as markers for “atherosclerosis” or “endothelial cell biology”, including “angiogenesis, vasodilation and -constriction”, coagulation, and platelet activation, by Qiagen RT^2^ Profiler PCR Arrays. The genes were plotted that were detected in our dataset were determined to be differentially expressed (FDR ≤ 0.05, log_2_-fold change ≥ 0.5 or ≤ -0.5).

### Cytokine detection

Several cytokines, such as CXCL11/I-TAC, IFNγ, IL-12p70, IL-1β, IL-23, IL-6, IL-8 and TNF-α, were measured in the frozen collected sample media by using the MILLIPLEX MAP Human High Sensitivity T-Cell Panel-Immunology Multiplex Assay. Cytokines were measured with a Milliplex Magpix instrument (Luminex, Merck KGaA, Darmstadt, GE). Analysis was performed according to the manufacturer’s instructions. Twenty-five microliters of the cell medium was used for the analysis. The calibration curves for each cytokine were calculated within the assay, and the detection ranges for each cytokine were different: CXCL11/I-TAC: 1.46–6000 pg/mL, IFNγ: 0.61–2500 pg/mL, IL-12p70: 0.49–2000 pg/mL, IL-1β: 0.49–2000 pg/mL, IL-23: 7.23–32,500 pg/mL, IL-6: 0.18–750 pg/mL, IL-8: 0.31–1250 pg/mL and TNF-α 0.43–1750 pg/mL. Cytokine concentrations were determined by fluorescence intensity, and fluorescence data were analyzed with Millipore Milliplex Analyst version 3.4 according to the manufacturer’s recommendations. Statistical analyses were performed with the calculated concentrations of each sample.

### Metabolomic sample preparation and untargeted GC/MS of sample media

Metabolomes were detected from the frozen collected sample media from three independent experiments per aerosol type. The derivatization method and GC/MS setup used were modified from the methods described previously Pink, Verma [103]. Then, 50 µL of the thawed collected sample media was added to 450 µL of a cold methanol/water mixture (88.9%, 11.1%) on ice. After vortexing, the sample was centrifuged for 5 min (18000 rcf, 4 °C). One hundred microliters were then transferred to a 1.5 mL GC vial and dried in a Speedvac. To obtain water-free samples, 100 µL of dichloromethane was added, and the samples were subsequently dried in a Speedvac. The extract was solubilized in 50 µL of a methoxyamine hydrochloride solution (20 mg/mL in pyridine) by sonication and incubated at 50 °C for 60 min before being placed on an orbital shaker at room temperature overnight. Afterwards, 25 µL of MSTFA (N-methyl-N-(trimethylsilyl) trifluoroacetamide) supplemented with 1% trimethylchlorosilane (TMCS) was added, and the mixture was further incubated for 60 min at 40 °C. For analysis, the samples were transferred to 100 µL inserts and frozen at − 80 °C until analysis. The derivatized metabolites were separated and semi-quantified by GC/MS analysis using an HP 8890/5977B GC/MS (Agilent Technologies, Waldbronn, Germany) instrument equipped with a 30 m × 320 μm (i.d.) Optima 5 column coated with a 5% phenyl/ 95% methylpolysolixane cross-linked stationary phase (0.25 μm film thickness; MACHEREY-NAGEL, Düren, Germany). Helium was used as the carrier gas at a flow rate of 1.5 mL/min. Two microliters of each sample were injected (injector temperature: 250 °C) in splitless mode with a solvent cutoff time of 6 min. The oven temperature was maintained at 80 °C for 10 min and then linearly increased at a rate of 5 °C/min up to 330 °C. The MS instrument was operated in electron impact ionization mode at 70 eV with the quadrupole temperature set at 150 °C and the source temperature set at 230 °C. Full scans were acquired by repetitive scanning over the mass range from 60 to 550 Da at a scan rate of 500 ms/scan.

### GC/MS spectrum and data analysis of sample media

A detailed description of the conversion of the GC/MS spectra, the database and the peak identification was previously described in Pink, Verma [104]. Briefly, the GC/MS spectra were evaluated by running the R package eRah [105], and peak identification was performed with the GOLM metabolome database (version 2011-11-21) [106] supplemented with data from NIST14 (NIST 14 Mass Spec Library and Search Programs – User Manual (sisweb.com)). Metabolome profiling was conducted in three independent experiments (*n* = 3). The resulting metabolites were annotated with the metabolite ID conversion tool of MetaboAnalyst 5.0 software and subjected to PCA and volcano plotting [107]. Moreover, the list of altered metabolites (log_2_FC ≤ -0.5/ ≥ 0.5; *p* ≤ 0.05) was uploaded alone or together with the RNASeq data (log_2_FC ≤ -0.5/ ≥ 0.5; *p* ≤ 0.05) in the Ingenuity Pathway Analysis (IPA, Qiagen) software for integrated analysis of canonical pathways.

## Electronic supplementary material

Below is the link to the electronic supplementary material.

Supplementary Material 1

Supplementary Material 2

Supplementary Material 3

Supplementary Material 4

## Data Availability

The sequencing data have been deposited in the NCBI´s Gene Expression Omnibus and are accessible through the GEO Series accession number GSE226350. Metabolome data is provided within the manuscript or supplementary information files.
