# Supplementary material for: The chemical composition of secondary organic aerosols regulates transcriptomic and metabolomic signaling in an epithelial-endothelial in vitro coculture
Source: Part Fibre Toxicol. 2024 Sep 19;21:38. doi: 10.1186/s12989-024-00600-x (PMC11411994; doi:10.1186/s12989-024-00600-x)
Supplement: Supplementary file 4 — Supplementary Material 4 [file 12989_2024_600_MOESM4_ESM.docx]

# **Supplemental Material**

**The Chemical Composition of Secondary Organic Aerosols Regulates Transcriptomic and Metabolomic Signaling in an Epithelial-Endothelial *In Vitro* Coculture**

Svenja Offer^1,2^, Sebastiano Di Bucchianico^1,2,3^*, Hendryk Czech^1,2^, Michal Pardo^4^, Jana Pantzke^1,2^, Christoph Bisig^1^, Eric Schneider^2,3^, Stefanie Bauer^1^, Elias J. Zimmermann^1,2^, Sebastian Oeder^1^, Elena Hartner^1,2^, Thomas Gröger^1,2^, Rasha Alsaleh^5^, Christian Kersch^5^, Till Ziehm^6^, Thorsten Hohaus^6^, Christopher P. Rüger^1,2,3^, Simone Schmitz-Spanke^5^, Jürgen Schnelle-Kreis^1^, Martin Sklorz^1^, Astrid Kiendler-Scharr^6†^, Yinon Rudich^4^ and Ralf Zimmermann^1,2,3^

*corresponding author: <mailto:dibucchianico@helmholtz-muenchen.de>

# **Table of Contents**

**Table S1.**  Deposition and toxicity results of co-culture exposed to SP, SOA_βPIN_-SP, and SOA_NAP_-SP.

**Table S2.** Top 5 VOC found in SOA_NAP_-SP and SOA_βPIN_-SP measured by PTR. Results are shown as the concentration [ppb] ± SD of n = 3 independent experiments.

**Table S3.** The most up- and downregulated genes in A549 cells after the exposure to SP compared to the CA Ctrl. Data are from n = 3 independent experiments.

**Table S4.** The most up- and downregulated genes in A549 cells after the exposure to SOA_βPIN_-SP compared to the CA Ctrl. Data are from n = 4 independent experiments.

**Table S5.** The most up- and downregulated genes in A549 cells after the exposure to SOA_NAP_-SP compared to the CA Ctrl. Data are from n = 4 independent experiments.

**Table S6.** The most up- and downregulated genes in EA.hy926 cells after the exposure to SP compared to the CA Ctrl. Data are from n = 3 independent experiments.

**Table S7.** The most up- and downregulated genes in EA.hy926 cells after the exposure to SOA_βPIN_-SP compared to the CA Ctrl. Data are from n = 4 independent experiments.

**Table S8.** The most up- and downregulated genes in EA.hy926 cells after the exposure to SOA_NAP_-SP compared to the CA Ctrl. Data are from n = 4 independent experiments.

**Figure S1.** Number of oxygen atoms (#O) vs double bond equivalents (DBE).

**Figure S2.** Differential expression analysis.

**Figure S3.** Volcano Plots.

**Table S1.** Deposition and toxicity results of co-culture exposed to SP, SOA_βPIN_-SP, and SOA_NAP_-SP. Co-cultures (A549/EA.hy926) were exposed 4 h in an ALI exposure system to unaged pure SP (CAST soot; 1mg m^-3^) or atmospheric photooxidation (aging) by OH radicals in a PAM (potential aerosol mass) reactor of SP (CAST soot; 1mg m^-3^) together with either naphthalene (4 mg m^-3^) or β-pinene (4 mg m^-3^), forming SOA_NAP_-SP and SOA_βPIN_-SP, respectively. Results are presented as mean values ± SEM of n independent experiments (Ctrl: n = 8, SP: n =4, SOA_βPIN_-SP: n = 4 and SOA_NAP_-SP: n = 5 for all dilutions). Angiogenic potential was calculated using the following equation $Angiogenic score=\left( \frac{\left( No. of sprouting cells \right)*1+\left( No. of connected cells \right)*2+\left( no. of polygons \right)*3}{Total number of cells} \right)+(0, 1 or 2)$. The addition of 0, 1 or 2 to the total value depends on the presence of a complex mesh. No complex mesh is represented by the addition of 0 points, for the existence of luminal structures consisting of 2-3 cell layers an addition of 1 point is necessary and for luminal structures greater than 3 cell layers an additional value of 2 is added.

|  | Ctrl | SP | | | | SOA_NAP_-SP | | | | SOA_βPIN_-SP | | | |
| --- | --- | --- | --- | --- | --- | --- | --- | --- | --- | --- | --- | --- | --- |
| Dilution |  | undiluted (1:1) | 1:3 | 1:10 | 1:30 | undiluted (1:1) | 1:3 | 1:10 | 1:30 | undiluted (1:1) | 1:3 | 1:10 | 1:30 |
| Deposition ng cm^-2^ | - | 9 ± 1 | ≈ 3 | ≈ 0.9 | ≈ 0.3 | 28 ± 2 | ≈ 9 | ≈ 3 | ≈ 0.9 | 17 ± 2 | ≈ 6 | ≈ 2 | ≈ 0.6 |
| LDH mU ml^-1^ | 38 ± 5 | 148 ± 50 | 57 ± 13 | 216 ± 69 | 101 ± 19 | 157 ± 40 | 56 ± 8 | 43 ± 14 | 59 ± 9 | 158 ± 32 | 68 ± 8 | 83 ± 19 | 118 ± 29 |
| MDA ng ml^-1^ | 6 ± 1 | 11 ± 5 | 8 ± 2 | 9 ± 3 | 8 ± 1 | 53 ± 8 | 27 ± 3 | 14 ± 2 | 10 ± 1 | 18 ± 6 | 12 ± 3 | 4 ± 2 | 7 ± 4 |
| % DNA breaks | **A549:**  4 ± 0.1 **EA.hy926:**  4 ± 0.1 | **A549:**  8 ± 0.2 **EA.hy926:**  4 ± 0.1 | **A549:**  7 ± 0.5  **EA.hy926:**  4 ± 0.1 | **A549:**  5 ± 0.6  **EA.hy926:**  4 ± 0.1 | **A549:**  5 ± 0.4  **EA.hy926:**  4 ± 0.2 | **A549:**  17 ± 0.8  **EA.hy926:**  8 ± 0.3 | **A549:**  19 ± 0.6 **EA.hy926:**  7 ± 0.4 | **A549:**  11 ± 1.5 **EA.hy926:**  5 ± 0.2 | **A549:**  15 ± 1.6 **EA.hy926:**  5 ± 0.2 | **A549:**  12 ± 1.6 **EA.hy926:**  4 ± 0.3 | **A549:**  9 ± 0.3 **EA.hy926:**  4 ± 0.1 | **A549:**  9 ± 1.2 **EA.hy926:**  4 ± 0.1 | **A549:**  6 ± 0.5 **EA.hy926:**  4 ± 0.05 |
| IL8 pg ml^-1^ | 340 ± 28 | 520 ± 41 | 490 ± 28 | 696 ± 53 | 430 ± 20 | 1199 ± 271 | 934 ± 47 | 824 ± 26 | 881 ± 26 | 878 ± 287 | 666 ± 22 | 651 ± 22 | 569 ± 32 |
| Angiogenic potential | 3.3 ± 0.3 | 3.4 ± 0.1 | - | - | - | 3.8 ± 0.2 | - | - | - | 3.0 ± 0.3 | - | - | - |

**Table S2.** Top 5 VOC found in SOA_NAP_-SP and SOA_βPIN_-SP measured by PTR. Results are shown as the concentration [ppb] ± SD of n = 3 independent experiments.

| **VOC** | **SOA_NAP_-SP** | | **SOA_βPIN_-SP** | |
| --- | --- | --- | --- | --- |
|  | **concentration [ppb]** | **SD [ppb]** | **concentration [ppb]** | **SD [ppb]** |
| C_10_H_8_: naphthalene | 96 | 3 | - | - |
| C_5_H_2_O_2_: unknown | 58 | 1 | - | - |
| CH_4_O: methanol | 37 | 4 | 59 | 2 |
| C_4_H_8_: butene | 24 | 12 | - | - |
| CH_2_O_2_: formic acid | 23 | 4 | 53 | 11 |
| C_3_H_6_O: aceton/propanal | - | - | 136 | 25 |
| CH_2_O: formaldehyde | - | - | 62 | 5 |
| C_2_H_4_O_2_: acetic acid | - | - | 29 | 7 |

**Table S3.** The most up- and downregulated regulated genes in A549 cells after the exposure to SP compared to the CA Ctrl. Data are from n = 3 independent experiments.

| **geneID** | **logFC** | **adjusted p-value** |
| --- | --- | --- |
| **upregulated** | | |
| *MALAT1* | 1.726874 | 0.000815 |
| *CXCL2* | 1.583143 | 0.001 |
| *PANK4* | 1.315595 | 0.003307 |
| *CENPJ* | 1.260884 | 0.005803 |
| *ZHX2* | 1.253002 | 0.008542 |
| *ABCG1* | 1.249019 | 0.050601 |
| *CCL2* | 1.179372 | 0.001601 |
| *PTGS2* | 1.147687 | 6.28E-05 |
| *OTUD3* | 1.132627 | 0.010739 |
| *EVA1C* | 1.102215 | 0.00083 |
| *VCAN* | 1.098653 | 0.000709 |
| *ID4* | 1.061741 | 0.160592 |
| *GLI2* | 1.050886 | 0.027379 |
| *MIR193BHG* | 1.048912 | 0.012676 |
| *PKD1* | 1.036434 | 0.006374 |
| *TGFBR3* | 1.028726 | 0.003335 |
| *KLHL29* | 1.001958 | 0.079726 |
| *MT.TM* | 0.998811 | 0.03458 |
| *CXCL1* | 0.998169 | 0.013377 |
| *SLC25A29* | 0.996663 | 0.002449 |
| *CXCL8* | 0.99312 | 0.007484 |
| *TNFSF15* | 0.979649 | 0.102952 |
| *CENPE* | 0.969073 | 0.022929 |
| *PSD4* | 0.950042 | 0.001707 |
| *OBSCN* | 0.949554 | 0.066008 |
| *FAM83A* | 0.947254 | 0.016929 |
| *CEMIP* | 0.938158 | 0.07641 |
| *DDX3X* | 0.92921 | 4.28E-05 |
| *GNAZ* | 0.928673 | 0.006323 |
| *SBK1* | 0.927897 | 0.043727 |
| *NR4A2* | 0.919582 | 0.018517 |
| *ZMAT4* | 0.91194 | 0.080409 |
| *RPS6KA5* | 0.900711 | 0.004342 |
| *ZC3H12A* | 0.898748 | 0.041137 |
| *RAI1* | 0.898525 | 0.023201 |
| *GBP2* | 0.890845 | 0.016656 |
| *CA5B* | 0.890525 | 0.021167 |
| *ANPEP* | 0.889393 | 0.002547 |
| *STRIP1* | 0.886868 | 0.00182 |
| *CGNL1* | 0.885679 | 0.038659 |
| *TP63* | 0.881408 | 0.011977 |
| *NFAT5* | 0.881314 | 0.002739 |
| *SLC38A2* | 0.872401 | 4.28E-05 |
| *GADD45G* | 0.871539 | 0.164248 |
| *THRAP3* | 0.86692 | 0.00271 |
| *RELT* | 0.864991 | 0.011409 |
| *GLUL* | 0.863651 | 0.000264 |
| *SLC19A3* | 0.85145 | 0.022211 |
| *CD58* | 0.849953 | 0.001707 |
| *TXNRD3* | 0.84787 | 0.054136 |
| *CEP72* | 0.837896 | 0.020701 |
| *XRCC2* | 0.834721 | 0.035325 |
| *ASPM* | 0.825095 | 0.004571 |
| *NFKBIA* | 0.824598 | 0.001896 |
| *SLC6A6* | 0.823164 | 0.003583 |
| *NFKBIZ* | 0.816951 | 0.052798 |
| *CCDC126* | 0.808719 | 0.015884 |
| *FHOD1* | 0.803894 | 0.000977 |
| *SYNE2* | 0.802326 | 0.02339 |
| *SPRED3* | 0.787355 | 0.039288 |
| **downregulated** | | |
| *SNAPC1* | -1.3039 | 0.000208 |
| *SERTAD4.AS1* | -1.4035 | 0.012441 |
| *AC005261.1* | -1.9164 | 0.001 |
| *PPP1R3C* | -1.29279 | 0.00029 |
| *ZNF416* | -1.29693 | 0.000709 |
| *EGR2* | -1.30926 | 0.100902 |
| *GJB3* | -1.31289 | 0.005931 |
| *ZNF350* | -1.31463 | 0.001985 |
| *GPNMB* | -1.33656 | 0.001896 |
| *EOMES* | -1.33794 | 0.00417 |
| *DOK7* | -1.34602 | 0.00091 |
| *ZNF280A* | -1.35252 | 0.058662 |
| *MMP10* | -1.36453 | 0.007484 |
| *WDR5B* | -1.36693 | 0.004794 |
| *LRIF1* | -1.36895 | 0.000447 |
| *ZNF112* | -1.37447 | 0.023326 |
| *BTG2* | -1.39376 | 4.28E-05 |
| *ZBED9* | -1.39662 | 0.003379 |
| *WDR66* | -1.41127 | 0.008045 |
| *ZNF674.AS1* | -1.41968 | 0.001326 |
| *RPL13AP20* | -1.42427 | 0.001896 |
| *KRT8P12* | -1.43139 | 0.002991 |
| *DLX4* | -1.43278 | 0.001 |
| *ZBED8* | -1.43932 | 0.005633 |
| *ZNF365* | -1.44852 | 0.002027 |
| *CCDC184* | -1.47979 | 0.000264 |
| *ZC3H10* | -1.48226 | 0.001304 |
| *ZNF551* | -1.49003 | 0.000264 |
| *TSPYL4* | -1.50734 | 0.001 |
| *MIR137HG* | -1.52348 | 0.005931 |
| *USP2* | -1.52417 | 0.06514 |
| *DHRS2* | -1.53289 | 0.023608 |
| *ANKRD1* | -1.54597 | 0.001327 |
| *ZNF548* | -1.55299 | 0.0019 |
| *ZSWIM3* | -1.55946 | 0.001777 |
| *OXTR* | -1.59136 | 0.000264 |
| *ZNF607* | -1.60588 | 0.000815 |
| *KRT80* | -1.63894 | 0.000264 |
| *HSPA1A* | -1.67719 | 0.001304 |
| *PEX12* | -1.72503 | 0.000315 |
| *NOG* | -1.74929 | 0.001448 |
| *CCDC121* | -1.79465 | 0.002711 |
| *PARS2* | -1.79761 | 0.003937 |
| *ACHE* | -1.83006 | 0.007979 |
| *ZNF585A* | -1.84484 | 0.004342 |
| *MAF* | -1.86926 | 0.005563 |
| *HSPA1B* | -1.97476 | 0.000977 |
| *RAB39B* | -1.98057 | 0.000264 |
| *PLEKHA8P1* | -2.00285 | 0.000754 |
| *HSPB8* | -2.03271 | 0.000264 |
| *CYP1A1* | -2.16866 | 0.001185 |
| *PIWIL2* | -2.23718 | 0.001238 |
| *AC084880.1* | -2.31532 | 0.003477 |
| *ALOXE3* | -2.42689 | 0.002882 |
| *PGF* | -2.61131 | 0.000281 |
| *HSPA1L* | -2.74475 | 0.00352 |
| *AL606500.1* | -2.89215 | 0.001707 |
| *ARL14* | -2.93301 | 0.00051 |
| *HIF1A.AS3* | -3.50537 | 0.000594 |
| *HSPA6* | -4.35587 | 0.00466 |

**Table S4.** The most up- and downregulated regulated genes in A549 cells after the exposure to SOA_βPIN_-SP compared to the CA Ctrl. Data are from n = 4 independent experiments.

| **geneID** | **logFC** | **adjusted p-value** | |
| --- | --- | --- | --- |
| **upregulated** | | | |
| *ABCG1* | 1.711652 | 0.001545 | |
| *CCL2* | 1.675815 | 5.82E-06 | |
| *PTGS2* | 1.659204 | 1.73E-07 | |
| *SOX21* | 1.592859 | 0.000883 | |
| *MALAT1* | 1.553169 | 0.000174 | |
| *SOX21.AS1* | 1.502238 | 0.003573 | |
| *CXCL2* | 1.40269 | 0.000256 | |
| *CEMIP* | 1.375044 | 0.001721 | |
| *TRPC6* | 1.358246 | 0.003762 | |
| *NFKBIA* | 1.326618 | 2.75E-06 | |
| *BCL6* | 1.31664 | 4.67E-06 | |
| *MUC5AC* | 1.312912 | 0.003164 | |
| *TGFBR3* | 1.296041 | 4.23E-05 | |
| *FCGBP* | 1.278223 | 0.005266 | |
| *PANK4* | 1.247646 | 0.000695 | |
| *SLC25A29* | 1.238214 | 3.97E-05 | |
| *RAI1* | 1.220649 | 0.000504 | |
| *EHF* | 1.216976 | 0.002754 | |
| *FGFBP1* | 1.195699 | 0.000959 | |
| *SYBU* | 1.194507 | 0.001693 | |
| *CENPE* | 1.18743 | 0.001036 | |
| *DDX3X* | 1.160409 | 1.98E-07 | |
| *TNFSF15* | 1.150758 | 0.01528 | |
| *KCNE4* | 1.147912 | 0.020496 | |
| *CA5B* | 1.143731 | 0.000554 | |
| *ID4* | 1.128255 | 0.050567 | |
| *CXCL1* | 1.126201 | 0.000963 | |
| *RPS6KA5* | 1.114728 | 7.05E-05 | |
| *ST8SIA4* | 1.108851 | 0.001045 | |
| *SLC6A6* | 1.108632 | 3.35E-05 | |
| *EVA1C* | 1.107602 | 5.81E-05 | |
| *ASPM* | 1.102271 | 4.08E-05 | |
| *TRIM31* | 1.085687 | 0.024213 | |
| *CENPJ* | 1.081146 | 0.003261 | |
| *MUC5B* | 1.079571 | 0.016405 | |
| *HS6ST1* | 1.074337 | 0.00035 | |
| *SLC7A2* | 1.06845 | 0.00024 | |
| *CARD6* | 1.047667 | 0.000183 | |
| *SDK2* | 1.0442 | 0.025107 | |
| *TNFSF9* | 1.039643 | 0.00042 | |
| *AXIN2* | 1.020417 | 0.000131 | |
| *SERINC5* | 1.007514 | 0.000303 | |
| *RNASE4* | 1.007152 | 0.009442 | |
| *RELL1* | 1.004625 | 0.000382 | |
| *PSD4* | 1.001862 | 0.000112 | |
| *KRT20* | 1.001083 | 0.089164 | |
| *PRRG4* | 0.998029 | 0.000512 | |
| *NFAT5* | 0.985788 | 0.000124 | |
| *CXCL8* | 0.984257 | 0.001429 | |
| *CHST12* | 0.981193 | 9.67E-05 | |
| *RAB40B* | 0.977567 | 2.22E-05 | |
| *USF3* | 0.976253 | 0.014103 | |
| *NR4A2* | 0.974091 | 0.002871 | |
| *UNC5D* | 0.966676 | 0.001604 | |
| *NKD1* | 0.965058 | 0.004909 | |
| *PKD1* | 0.96376 | 0.001876 | |
| *ATG2A* | 0.941814 | 0.000166 | |
| *TOX3* | 0.939602 | 0.001764 | |
| *ZHX2* | 0.931498 | 0.013523 | |
| *PLA2G4A* | 0.929991 | 1.15E-05 | |
| **downregulated** | | | |
| *SERPINE1* | -2.153 | | 4.72E-05 |
| *ZNF112* | -2.411 | | 0.000144 |
| *ANKRD1* | -3.161 | | 8.61E-07 |
| *RPL13AP20* | -1.8332 | | 2.02E-05 |
| *FOXJ1* | -1.8742 | | 0.003042 |
| *MIR137HG* | -2.0934 | | 7.46E-05 |
| *ALOXE3* | -3.1289 | | 4.21E-05 |
| *ARL14* | -3.2884 | | 8.32E-06 |
| *NOG* | -3.7219 | | 1.54E-06 |
| *AMOTL2* | -1.78551 | | 7.86E-06 |
| *SERTAD4.AS1* | -1.79039 | | 0.000306 |
| *CCN2* | -1.81964 | | 1.30E-05 |
| *SP6* | -1.82186 | | 0.000211 |
| *ANKRD37* | -1.82237 | | 0.000632 |
| *ZBED8* | -1.83798 | | 0.000101 |
| *SERTAD4* | -1.84412 | | 0.000131 |
| *PPP1R3C* | -1.84605 | | 8.20E-07 |
| *GATA3* | -1.86225 | | 2.02E-05 |
| *DLX4* | -1.86534 | | 7.86E-06 |
| *TSPYL4* | -1.86881 | | 1.23E-05 |
| *TAGLN* | -1.88718 | | 0.002976 |
| *ZNF365* | -1.91097 | | 2.38E-05 |
| *KLHDC7A* | -1.91719 | | 1.73E-07 |
| *PDGFB* | -1.95167 | | 7.00E-07 |
| *RNF225* | -1.95502 | | 0.00089 |
| *ZNF280A* | -1.96294 | | 0.001855 |
| *ZNF350* | -1.96748 | | 9.41E-06 |
| *RBM12B* | -1.96792 | | 1.54E-06 |
| *PEX12* | -2.01673 | | 4.33E-06 |
| *RAB39B* | -2.02994 | | 6.86E-06 |
| *AC026803.2* | -2.03343 | | 0.003625 |
| *ZNF134* | -2.06474 | | 8.20E-07 |
| *TUFT1* | -2.09828 | | 1.40E-06 |
| *NCF2* | -2.10193 | | 0.000152 |
| *ZNF548* | -2.10674 | | 2.00E-05 |
| *AC005261.1* | -2.12646 | | 2.78E-05 |
| *CCDC121* | -2.14281 | | 6.57E-05 |
| *MARCHF4* | -2.14381 | | 0.000355 |
| *AL133346.1* | -2.15218 | | 3.31E-05 |
| *ZNF551* | -2.15319 | | 8.20E-07 |
| *HSPA1A* | -2.16279 | | 1.42E-05 |
| *USP43* | -2.23243 | | 1.65E-05 |
| *EGR3* | -2.23555 | | 0.008125 |
| *OXTR* | -2.29594 | | 8.20E-07 |
| *USP2* | -2.32491 | | 0.002129 |
| *HSPA1B* | -2.39076 | | 1.47E-05 |
| *AL606500.1* | -2.43789 | | 0.000295 |
| *MAF* | -2.46388 | | 0.000118 |
| *HSPB8* | -2.56762 | | 1.54E-06 |
| *KRT80* | -2.57597 | | 2.33E-07 |
| *CYP1A1* | -2.76749 | | 1.15E-05 |
| *EGR2* | -2.88937 | | 0.000879 |
| *PGF* | -2.97679 | | 4.33E-06 |
| *GDF6* | -2.97914 | | 6.67E-05 |
| *AC084880.1* | -3.17284 | | 7.03E-05 |
| *PIWIL2* | -3.24919 | | 5.82E-06 |
| *ACHE* | -3.68617 | | 2.26E-05 |
| *HSPA1L* | -3.73604 | | 7.12E-05 |
| *HIF1A.AS3* | -6.12587 | | 1.98E-07 |
| *HSPA6* | -6.27223 | | 0.00037 |

**Table S5.** The most up- and downregulated regulated genes in A549 cells after the exposure to SOA_NAP_-SP compared to the CA Ctrl. Data are from n = 4 independent experiments.

| **geneID** | **logFC** | **adjusted p-value** |
| --- | --- | --- |
| **upregulated** | | |
| *CXCL2* | 3.037392 | 8.89E-08 |
| *MT.TM* | 2.714405 | 9.48E-07 |
| *FCGBP* | 2.501709 | 9.23E-06 |
| *CXCL8* | 2.430198 | 1.59E-07 |
| *MUC5AC* | 2.395647 | 1.11E-05 |
| *CEACAM5* | 2.37897 | 0.000172 |
| *MUC5B* | 2.354326 | 2.37E-05 |
| *MT.TL1* | 2.236635 | 2.17E-05 |
| *PTGS2* | 2.211106 | 1.43E-09 |
| *ID4* | 2.181555 | 0.000384 |
| *BPIFB1* | 2.052461 | 0.000328 |
| *CEACAM1* | 2.016902 | 3.71E-06 |
| *CXCL1* | 1.979493 | 3.16E-06 |
| *CST1* | 1.97579 | 0.000271 |
| *GSTA1* | 1.968333 | 0.00071 |
| *MT.TV* | 1.928918 | 0.000324 |
| *NR4A2* | 1.89908 | 5.01E-06 |
| *GJB1* | 1.894745 | 0.000134 |
| *MALAT1* | 1.880153 | 2.12E-05 |
| *CXCL3* | 1.850711 | 1.46E-07 |
| *GADD45G* | 1.827697 | 0.00045 |
| *TENT5C* | 1.823471 | 0.000224 |
| *CREB3L1* | 1.805691 | 2.48E-05 |
| *KRT20* | 1.782951 | 0.002352 |
| *RASD1* | 1.675818 | 0.000806 |
| *FOS* | 1.658903 | 0.000126 |
| *NOX1* | 1.647398 | 0.000838 |
| *CEMIP* | 1.608103 | 0.000313 |
| *DUSP10* | 1.561722 | 3.76E-08 |
| *SLC13A2* | 1.548329 | 0.000101 |
| *FOXA3* | 1.540406 | 3.28E-05 |
| *TFF1* | 1.534193 | 0.000293 |
| *LGALS4* | 1.522422 | 0.000593 |
| *HLA.DMB* | 1.503548 | 6.82E-05 |
| *CP* | 1.498592 | 0.000499 |
| *PANK4* | 1.497614 | 8.57E-05 |
| *TJP3* | 1.47382 | 1.41E-05 |
| *TRIM31* | 1.471018 | 0.002305 |
| *CEACAM6* | 1.450298 | 0.000277 |
| *TOX3* | 1.448933 | 2.29E-05 |
| *ZC3H12A* | 1.435937 | 0.000254 |
| *TM4SF5* | 1.423919 | 0.001534 |
| *SNAI1* | 1.419746 | 0.009061 |
| *ZFP36* | 1.406232 | 9.08E-07 |
| *MT.TF* | 1.4052 | 0.001372 |
| *ABCG1* | 1.398368 | 0.005171 |
| *SOX21* | 1.395835 | 0.001853 |
| *ADCY5* | 1.395165 | 2.72E-05 |
| *WNT4* | 1.390257 | 4.82E-05 |
| *ST6GALNAC1* | 1.387971 | 0.001643 |
| *EPS8L3* | 1.367636 | 0.000543 |
| *ERBB3* | 1.366771 | 0.000138 |
| *PNRC1* | 1.359035 | 6.74E-08 |
| *VIL1* | 1.35745 | 0.000164 |
| *GLUL* | 1.355327 | 1.08E-07 |
| *ACSL5* | 1.354257 | 0.038414 |
| *CORO2A* | 1.340598 | 0.000476 |
| *PKD1* | 1.340155 | 7.27E-05 |
| *BCL6* | 1.33112 | 2.63E-06 |
| TM4SF20 | 1.328446 | 1.27E-05 |
| **downregulated** | | |
| *ZBED8* | -1.82658 | 7.91E-05 |
| *COL22A1* | -1.83562 | 0.000312 |
| *PLEKHA8P1* | -1.85468 | 3.19E-05 |
| *ZNF529* | -1.85691 | 2.14E-06 |
| *TSPYL4* | -1.86646 | 7.02E-06 |
| *ZNF773* | -1.8715 | 1.43E-05 |
| *SERPINE1* | -1.87272 | 0.000119 |
| *MIR137HG* | -1.88046 | 0.000101 |
| *IGFL1* | -1.89831 | 0.001289 |
| *NCF2* | -1.90127 | 0.000185 |
| *SPARC* | -1.90362 | 0.000358 |
| *ZNF550* | -1.90393 | 7.64E-06 |
| *ZNF766* | -1.90665 | 6.64E-06 |
| *RBP1* | -1.91275 | 0.000871 |
| *GSPT2* | -1.91977 | 3.65E-06 |
| *BBS12* | -1.92483 | 0.000128 |
| *PIWIL2* | -1.93104 | 0.00011 |
| *ACHE* | -1.93263 | 0.000427 |
| *MOB3B* | -1.958 | 1.50E-05 |
| *PEX12* | -1.95798 | 2.95E-06 |
| *NPTX1* | -1.97555 | 0.000146 |
| *HOXA1* | -1.99228 | 1.73E-06 |
| *KRT80* | -1.99741 | 1.44E-06 |
| *ZNF549* | -2.00063 | 3.65E-06 |
| *ZNF134* | -2.00567 | 4.32E-07 |
| *ALOXE3* | -2.00831 | 0.000505 |
| *ZNF112* | -2.02456 | 0.000306 |
| *ZNF350* | -2.03749 | 5.40E-06 |
| *ADAM19* | -2.03866 | 7.25E-06 |
| *ZSWIM3* | -2.05855 | 1.16E-05 |
| *ZNF674.AS1* | -2.08973 | 3.88E-06 |
| *HSPA1L* | -2.09607 | 0.000525 |
| *ZNF607* | -2.11076 | 4.09E-06 |
| *CCDC121* | -2.11965 | 4.89E-05 |
| *NEXN* | -2.14467 | 0.000153 |
| *NIPAL4* | -2.1503 | 6.67E-05 |
| *ZNF614* | -2.15475 | 4.38E-05 |
| *PDGFB* | -2.16561 | 1.52E-07 |
| *ZNF229* | -2.18618 | 3.75E-05 |
| *OXTR* | -2.19283 | 6.97E-07 |
| *SPOCK1* | -2.30189 | 1.01E-05 |
| *SERTAD4* | -2.32965 | 2.38E-05 |
| *R3HDML* | -2.33605 | 0.001048 |
| *RAB39B* | -2.39685 | 1.54E-06 |
| *ZNF551* | -2.41114 | 1.85E-07 |
| *AC005261.1* | -2.41276 | 7.33E-06 |
| *USP2* | -2.58681 | 0.000866 |
| *PARS2* | -2.59896 | 2.17E-05 |
| *MAF* | -2.70963 | 4.36E-05 |
| *ZNF14* | -2.79906 | 4.51E-06 |
| *ZNF365* | -2.85935 | 1.54E-06 |
| *HSPA6* | -2.90216 | 0.000697 |
| *TAGLN* | -2.92609 | 0.000248 |
| *ZNF548* | -2.92961 | 2.09E-06 |
| *AL606500.1* | -2.95202 | 7.29E-05 |
| *MARCHF4* | -3.1311 | 2.88E-05 |
| *HIF1A.AS3* | -3.13935 | 4.29E-05 |
| *GDF6* | -3.44827 | 2.11E-05 |
| *NOG* | -3.46676 | 1.47E-06 |

**Table S6.** The most up- and downregulated regulated genes in EA.hy926 cells after the exposure to SP compared to the CA Ctrl. Data are from n = 3 independent experiments.

| **geneID** | **logFC** | **adjusted p-value** |
| --- | --- | --- |
| **upregulated** | | |
| *AC083829.1* | 4.155723 | 1.51E-06 |
| *MAFA* | 2.799742 | 0.00183 |
| *HS3ST1* | 2.716182 | 8.69E-07 |
| *CXCL8* | 2.708229 | 0.000226 |
| *ERRFI1* | 2.501227 | 4.15E-09 |
| *BIRC3* | 2.490343 | 0.000896 |
| *SERPINB2* | 2.446455 | 0.000225 |
| *NR4A2* | 2.371984 | 1.87E-05 |
| *CXCL3* | 2.218002 | 0.001202 |
| *ZNF114* | 2.188773 | 0.000353 |
| *CCDC68* | 2.057418 | 3.36E-06 |
| *GEM* | 1.974138 | 2.60E-06 |
| *NFKBIZ* | 1.917922 | 0.001249 |
| *FOSB* | 1.913324 | 1.99E-05 |
| *RELN* | 1.867376 | 0.004769 |
| *AREG* | 1.843756 | 4.30E-05 |
| *TNFAIP8L3* | 1.841966 | 5.72E-05 |
| *NR4A1* | 1.836336 | 0.000658 |
| *ASPHD2* | 1.821658 | 2.59E-05 |
| *TNFRSF10D* | 1.816647 | 1.51E-06 |
| *AC110792.4* | 1.795647 | 1.98E-05 |
| *FST* | 1.717041 | 4.89E-05 |
| *IL11* | 1.713195 | 0.000501 |
| *ATF3* | 1.703983 | 8.69E-07 |
| *IER3* | 1.646687 | 4.89E-06 |
| *ANGPT2* | 1.627331 | 3.85E-05 |
| *ZC3H12A* | 1.611727 | 0.009311 |
| *NAV3* | 1.588979 | 1.30E-05 |
| *DUSP8* | 1.547268 | 5.54E-05 |
| *BAZ2B* | 1.526336 | 0.000751 |
| *IPMK* | 1.511934 | 6.76E-06 |
| *CXCL1* | 1.471041 | 0.015194 |
| *KDM7A* | 1.470986 | 0.001632 |
| *NFKBIA* | 1.466104 | 3.37E-07 |
| *CEMIP* | 1.464455 | 0.000401 |
| *KITLG* | 1.45042 | 1.85E-06 |
| *DHRS9* | 1.447903 | 0.000653 |
| *PGM2L1* | 1.422472 | 3.37E-07 |
| *PPP1R15A* | 1.4029 | 1.44E-05 |
| *DGKD* | 1.400991 | 2.72E-05 |
| *GPR3* | 1.393731 | 0.001072 |
| *IL6* | 1.390375 | 0.00291 |
| *RASSF8* | 1.387759 | 5.58E-05 |
| *KLF4* | 1.374376 | 0.00111 |
| *GCOM1* | 1.36944 | 0.000441 |
| *GRAMD1B* | 1.366772 | 1.81E-06 |
| *SPRY4* | 1.345216 | 1.87E-05 |
| *STC1* | 1.334729 | 0.000407 |
| *FGF16* | 1.329184 | 0.000653 |
| *NEDD9* | 1.326628 | 0.001198 |
| *PNRC1* | 1.325798 | 1.99E-06 |
| *EGR1* | 1.316558 | 0.051048 |
| *SLC4A7* | 1.316001 | 8.42E-06 |
| *NR4A3* | 1.30822 | 0.004349 |
| *TMEM217* | 1.307328 | 0.006021 |
| *OGT* | 1.304751 | 1.81E-06 |
| *FOS* | 1.29906 | 0.01729 |
| *FILIP1L* | 1.295335 | 0.000252 |
| *HDAC9* | 1.292203 | 7.80E-05 |
| *RELB* | 1.284639 | 0.000225 |
| **downregulated** | | |
| *THNSL1* | -1.1648 | 0.001076 |
| *CDCA7* | -1.2016 | 3.24E-05 |
| *SLC30A1* | -1.2027 | 0.000967 |
| *ELMSAN1* | -1.2156 | 3.49E-06 |
| *GIMAP1* | -1.2602 | 3.41E-06 |
| *ZNF449* | -2.4318 | 7.80E-05 |
| *HOXA1* | -1.14742 | 0.000711 |
| *CCDC9B* | -1.15016 | 0.000441 |
| *ZNF260* | -1.15035 | 7.80E-05 |
| *SLX4IP* | -1.15419 | 0.000707 |
| *ZC3H10* | -1.15659 | 0.000453 |
| *ZNF780A* | -1.16326 | 0.000103 |
| *PRKCQ.AS1* | -1.16335 | 0.000965 |
| *SESN1* | -1.16775 | 2.80E-06 |
| *TRAPPC2* | -1.17789 | 7.80E-05 |
| *TMEM236* | -1.18104 | 0.001129 |
| *TBC1D2B* | -1.19698 | 7.79E-05 |
| *CCRL2* | -1.19707 | 0.000117 |
| *SH2D3C* | -1.19766 | 0.000523 |
| *INKA2* | -1.20918 | 6.55E-05 |
| *AC015813.6* | -1.21189 | 0.001051 |
| *ZNF33B* | -1.21754 | 0.00012 |
| *ATOH8* | -1.21832 | 0.000441 |
| *ZNF675* | -1.22466 | 9.72E-05 |
| *NRAV* | -1.23018 | 0.005128 |
| *ZNF777* | -1.25291 | 7.00E-05 |
| *ZNF772* | -1.26271 | 0.000262 |
| *TIGD6* | -1.27568 | 0.000697 |
| *NEU3* | -1.29679 | 3.04E-05 |
| *FAM110D* | -1.29762 | 5.54E-05 |
| *LENG9* | -1.29964 | 3.85E-05 |
| *HMOX1* | -1.30916 | 0.051447 |
| *ZNF280B* | -1.30935 | 0.000192 |
| *HARBI1* | -1.32184 | 0.001152 |
| *SLC25A34.AS1* | -1.32811 | 0.000492 |
| *HOXA11* | -1.32838 | 0.000195 |
| *MEIS1* | -1.32882 | 0.001243 |
| *MT1X* | -1.33049 | 0.036423 |
| *SYNPO* | -1.33291 | 4.49E-06 |
| *GIMAP6* | -1.33473 | 2.06E-06 |
| *SP6* | -1.33968 | 1.99E-06 |
| *FANCF* | -1.35115 | 0.00055 |
| *APLN* | -1.41507 | 2.39E-06 |
| *PARS2* | -1.43905 | 0.000151 |
| *ZNF587B* | -1.44534 | 4.94E-05 |
| *PUS10* | -1.47422 | 5.17E-05 |
| *ZNF678* | -1.48304 | 0.001071 |
| *FAM50B* | -1.49464 | 7.87E-05 |
| *TRAC* | -1.49723 | 0.000225 |
| *ZNF512* | -1.51183 | 7.77E-05 |
| *ZNF619* | -1.53029 | 7.89E-05 |
| *AJUBA* | -1.53449 | 1.81E-06 |
| *LENG8* | -1.53501 | 4.75E-06 |
| *FAM43A* | -1.63743 | 1.51E-06 |
| *WDR5B* | -1.69755 | 0.000512 |
| *C1orf74* | -1.70594 | 1.98E-05 |
| *PDGFB* | -1.76897 | 4.49E-06 |
| *PLPP6* | -2.01588 | 2.19E-05 |
| *SLC22A5* | -2.17668 | 3.60E-05 |
| *OSBPL7* | -2.33032 | 0.000148 |

**Table S7.** The most up- and downregulated regulated genes in EA.hy926 cells after the exposure to SOA_βPIN_-SP compared to the CA Ctrl. Data are from n = 4 independent experiments.

| **geneID** | **logFC** | **adjusted p-value** |
| --- | --- | --- |
| **upregulated** | | |
| *AC083829.1* | 4.311127 | 2.52E-07 |
| *MAFA* | 2.979898 | 0.00055 |
| *CXCL8* | 2.847561 | 5.76E-05 |
| *NR4A2* | 2.79723 | 1.01E-06 |
| *HS3ST1* | 2.747601 | 1.99E-07 |
| *ERRFI1* | 2.658324 | 4.38E-10 |
| *FOSB* | 2.612534 | 2.41E-07 |
| *SERPINB2* | 2.41983 | 0.000125 |
| *EGR1* | 2.376086 | 0.000419 |
| *GEM* | 2.364985 | 1.26E-07 |
| *CXCL3* | 2.3138 | 0.000473 |
| *NR4A1* | 2.274063 | 3.40E-05 |
| *RELN* | 2.242656 | 0.000551 |
| *MT.TM* | 2.148978 | 0.008695 |
| *AC110792.4* | 2.112097 | 1.05E-06 |
| *CCDC68* | 2.071948 | 1.05E-06 |
| *IL6* | 2.03661 | 4.09E-05 |
| *BIRC3* | 2.033777 | 0.003333 |
| *NEDD9* | 2.019424 | 8.41E-06 |
| *ATF3* | 1.97293 | 4.85E-08 |
| *ZNF114* | 1.931421 | 0.000625 |
| *ZC3H12A* | 1.908926 | 0.001551 |
| *FOS* | 1.89597 | 0.000576 |
| *IER3* | 1.843875 | 4.40E-07 |
| *IL11* | 1.822857 | 0.000142 |
| *CXCL1* | 1.805761 | 0.002117 |
| *TNFRSF10D* | 1.803695 | 3.47E-07 |
| *ASPHD2* | 1.799203 | 1.10E-05 |
| *TNFAIP3* | 1.718971 | 8.47E-07 |
| *TNFAIP8L3* | 1.698786 | 6.41E-05 |
| *AREG* | 1.661398 | 5.74E-05 |
| *NFATC2* | 1.639673 | 0.000578 |
| *KLF4* | 1.597726 | 0.000139 |
| *PPP1R15A* | 1.582383 | 1.03E-06 |
| *IL24* | 1.564141 | 0.000863 |
| *PNRC1* | 1.560545 | 1.15E-07 |
| *IL1A* | 1.558557 | 0.000489 |
| *SPRY4* | 1.547126 | 1.20E-06 |
| *NR4A3* | 1.537494 | 0.000625 |
| *FST* | 1.511406 | 6.55E-05 |
| *NFKBIZ* | 1.51125 | 0.005398 |
| *HDAC9* | 1.470844 | 8.65E-06 |
| *TMEM217* | 1.46571 | 0.001422 |
| *IPMK* | 1.444614 | 3.60E-06 |
| *SLC4A7* | 1.417504 | 1.30E-06 |
| *TIAM2* | 1.413368 | 0.000436 |
| *NAV3* | 1.395022 | 1.83E-05 |
| *NFKBIA* | 1.394887 | 1.15E-07 |
| *FGF16* | 1.390915 | 0.000224 |
| *GRAMD1B* | 1.386677 | 3.47E-07 |
| *HBEGF* | 1.372683 | 9.35E-07 |
| *DUSP8* | 1.350539 | 0.000102 |
| *GRASP* | 1.348645 | 6.27E-05 |
| *FILIP1L* | 1.341426 | 7.22E-05 |
| *DHRS9* | 1.341202 | 0.000762 |
| *KITLG* | 1.336712 | 1.01E-06 |
| *GPR3* | 1.315922 | 0.001006 |
| *RELB* | 1.306519 | 8.60E-05 |
| *ADAMTS1* | 1.300273 | 2.35E-05 |
| *NUAK2* | 1.291039 | 2.78E-07 |
| **downregulated** | | |
| *ZNF449* | -1.843 | 0.000272 |
| *CDCA7* | -1.2951 | 6.27E-06 |
| *JRKL* | -1.22595 | 0.000251 |
| *DEXI* | -1.22673 | 0.000253 |
| *SP6* | -1.23664 | 1.19E-06 |
| *ZNF133* | -1.24577 | 0.005832 |
| *ZNF488* | -1.24981 | 1.72E-06 |
| *MEIS1* | -1.25197 | 0.000872 |
| *LIMD1* | -1.25512 | 4.20E-05 |
| *RSRP1* | -1.25902 | 0.005393 |
| *ZNF319* | -1.25965 | 1.91E-06 |
| *AC012313.1* | -1.26587 | 0.000271 |
| *NMNAT1* | -1.27019 | 0.000243 |
| *ZNF777* | -1.27672 | 2.35E-05 |
| *AC007906.2* | -1.28309 | 0.004033 |
| *TMCC3* | -1.28376 | 9.93E-06 |
| *CAVIN2* | -1.28521 | 1.10E-05 |
| *SH2D3C* | -1.29506 | 0.000161 |
| *TMEM140* | -1.29604 | 0.000171 |
| *SETD1B* | -1.31489 | 1.34E-05 |
| *ZNF561* | -1.31695 | 2.79E-07 |
| *NRAV* | -1.33044 | 0.001876 |
| *PTCH1* | -1.33134 | 9.70E-05 |
| *CCRL2* | -1.33476 | 2.22E-05 |
| *FAM78A* | -1.35109 | 3.42E-05 |
| *ZNF512* | -1.35245 | 6.88E-05 |
| *FANCF* | -1.35878 | 0.000272 |
| *FAM110D* | -1.36292 | 1.43E-05 |
| *NEU3* | -1.37149 | 6.25E-06 |
| *BEND3P1* | -1.37444 | 0.00084 |
| *PEX12* | -1.37659 | 1.90E-06 |
| *AJM1* | -1.37769 | 0.000716 |
| *TBC1D2B* | -1.38045 | 8.77E-06 |
| *KBTBD7* | -1.38117 | 0.003405 |
| *ZNF619* | -1.38415 | 7.21E-05 |
| *ZNF780A* | -1.38847 | 9.94E-06 |
| *LENG8* | -1.39358 | 3.74E-06 |
| *ZNF234* | -1.39373 | 7.62E-05 |
| *SYNPO* | -1.40591 | 7.75E-07 |
| *ZNF260* | -1.41311 | 6.25E-06 |
| *PARS2* | -1.42743 | 7.62E-05 |
| *ELMSAN1* | -1.42955 | 2.52E-07 |
| *SLC22A5* | -1.45347 | 0.000282 |
| *TMEM236* | -1.45833 | 0.000109 |
| *HARBI1* | -1.48048 | 0.000241 |
| *GIMAP6* | -1.59335 | 1.72E-07 |
| *AJUBA* | -1.63989 | 2.41E-07 |
| *HOXA11* | -1.70284 | 1.10E-05 |
| *PDGFB* | -1.71872 | 1.66E-06 |
| *PLPP6* | -1.71999 | 2.70E-05 |
| *CCDC9B* | -1.73694 | 8.10E-06 |
| *TRAC* | -1.74363 | 2.83E-05 |
| *APLN* | -1.75277 | 1.07E-07 |
| *C1orf74* | -1.77178 | 5.26E-06 |
| *WDR5B* | -1.81892 | 0.000163 |
| *HIC1* | -1.82496 | 9.60E-06 |
| *FAM50B* | -1.83965 | 6.37E-06 |
| *FAM43A* | -1.84699 | 1.15E-07 |
| *ZNF587B* | -1.86981 | 2.18E-06 |
| *OSBPL7* | -2.44458 | 2.25E-05 |

**Table S8.** The most up- and downregulated regulated genes in EA.hy926 cells after the exposure to SOA_NAP_-SP compared to the CA Ctrl. Data are from n = 4 independent experiments.

| **geneID** | **logFC** | **adjusted p-value** |
| --- | --- | --- |
| **upregulated** | | |
| *AC083829.1* | 5.000174723 | 9.85E-09 |
| *CXCL8* | 4.25192225 | 2.65E-07 |
| *CXCL3* | 3.983743173 | 2.64E-07 |
| *NR4A2* | 3.740103 | 1.06E-08 |
| *MAFA* | 3.359055 | 8.23E-05 |
| *FOS* | 3.334130184 | 5.00E-07 |
| *FOSB* | 3.282091977 | 5.51E-09 |
| *RELN* | 3.256667 | 4.81E-06 |
| *MT.TM* | 3.251865 | 0.000102 |
| *EGR1* | 3.184505689 | 7.51E-06 |
| *SERPINB2* | 3.157645 | 2.91E-06 |
| *NR4A1* | 3.059207 | 5.12E-07 |
| *ZC3H12A* | 3.015627 | 6.87E-06 |
| *GEM* | 3.009313414 | 3.39E-09 |
| *BIRC3* | 2.921679446 | 5.16E-05 |
| *ERRFI1* | 2.905634943 | 8.10E-11 |
| *NFKBIZ* | 2.873883 | 4.42E-06 |
| *NR4A3* | 2.754276 | 4.30E-07 |
| *HS3ST1* | 2.741032961 | 4.94E-08 |
| *CXCL1* | 2.712833328 | 1.71E-05 |
| *AC110792.4* | 2.657751152 | 1.90E-08 |
| *IL6* | 2.566278 | 1.25E-06 |
| *IL11* | 2.54634 | 1.52E-06 |
| *AREG* | 2.528098836 | 1.55E-07 |
| *ATF3* | 2.496923918 | 1.26E-09 |
| *FST* | 2.447678951 | 1.10E-07 |
| *CXCL2* | 2.354367069 | 0.005497 |
| *IL24* | 2.342867 | 5.63E-06 |
| *PPP1R15A* | 2.287658 | 6.15E-09 |
| *TNFRSF10D* | 2.286723 | 7.44E-09 |
| *IER3* | 2.253375 | 1.31E-08 |
| *CCDC68* | 2.244319108 | 1.34E-07 |
| *IL1A* | 2.172789 | 6.55E-06 |
| *PNRC1* | 2.131236 | 1.26E-09 |
| *ID2* | 2.036485 | 1.77E-06 |
| *SPRY4* | 1.997346 | 1.85E-08 |
| *TNFAIP8L3* | 1.966622 | 5.35E-06 |
| *TNFAIP3* | 1.949192 | 5.54E-08 |
| *ZNF114* | 1.947363 | 0.000321 |
| *GRASP* | 1.943121479 | 3.83E-07 |
| *DLX2* | 1.923899835 | 1.16E-05 |
| *KLF4* | 1.903569 | 9.22E-06 |
| *HBEGF* | 1.883627089 | 9.30E-09 |
| *NAV3* | 1.824645 | 3.16E-07 |
| *IPMK* | 1.821175 | 8.09E-08 |
| *KDM7A* | 1.799004 | 6.96E-05 |
| *TMEM217* | 1.77242 | 0.000132 |
| *STC1* | 1.748868 | 4.87E-06 |
| *FGF16* | 1.728690338 | 9.73E-06 |
| *DUSP8* | 1.723759749 | 2.88E-06 |
| *ANGPT2* | 1.710329929 | 3.55E-06 |
| *KITLG* | 1.669029 | 2.45E-08 |
| *NUAK2* | 1.655585 | 5.31E-09 |
| *HDAC9* | 1.652107007 | 8.31E-07 |
| *GRAMD1B* | 1.627407624 | 1.57E-08 |
| *ASPHD2* | 1.625878216 | 1.52E-05 |
| *IL1B* | 1.615925 | 0.007747 |
| *ARC* | 1.599615179 | 1.52E-06 |
| *DGKD* | 1.55422364 | 1.15E-06 |
| *NFKBIA* | 1.545426 | 9.30E-09 |
| **downregulated** | | |
| *JRKL* | -1.62286 | 1.03E-05 |
| *SYNPO* | -1.72408 | 2.57E-08 |
| *SETD1B* | -1.75441 | 3.61E-07 |
| *SLC22A5* | -1.97114 | 1.02E-05 |
| *ZBTB26* | -1.97359 | 6.99E-07 |
| *INAFM2* | -1.609163 | 1.90E-08 |
| *MAP3K1* | -1.609714 | 0.000375 |
| *ZNF107* | -1.611959 | 8.79E-06 |
| *ZNF329* | -1.629592 | 1.93E-05 |
| *LENG8* | -1.631159 | 2.78E-07 |
| *PIGM* | -1.643241 | 4.43E-06 |
| *TMEM204* | -1.646047 | 2.58E-07 |
| *ZNF780A* | -1.651369 | 7.83E-07 |
| *MTERF1* | -1.660213 | 3.78E-08 |
| *KBTBD6* | -1.668493 | 3.89E-07 |
| *TMEM140* | -1.717508 | 6.55E-06 |
| *TMCC3* | -1.721451 | 2.58E-07 |
| *ZNF234* | -1.730863 | 4.94E-06 |
| *ZNF273* | -1.753014 | 6.03E-06 |
| *RBM12B* | -1.786838 | 1.71E-08 |
| *ZNF449* | -1.795481 | 0.000174 |
| *JRK* | -1.797466 | 1.71E-08 |
| *SESN1* | -1.803937 | 5.97E-09 |
| *ZNF678* | -1.834671 | 4.73E-05 |
| *TMEM177* | -1.840476 | 9.30E-09 |
| *ZC3H10* | -1.890753 | 1.56E-06 |
| *ZNF619* | -1.906098 | 1.97E-06 |
| *INKA2* | -1.913959 | 1.81E-07 |
| *NEU3* | -2.037012 | 5.99E-08 |
| *PLPP6* | -2.147494 | 1.75E-06 |
| *KBTBD7* | -2.164308 | 7.29E-05 |
| *PARS2* | -2.171566 | 1.01E-06 |
| *OSBPL7* | -2.291152 | 2.23E-05 |
| *WDR5B* | -2.382379 | 8.53E-06 |
| *RTL10* | -2.440251 | 5.05E-08 |
| *PDGFB* | -2.527971 | 1.85E-08 |
| *C22orf34* | -1.6319393 | 4.76E-07 |
| *HCG11* | -1.62049906 | 1.15E-06 |
| *AJUBA* | -1.97948665 | 9.30E-09 |
| *FAM110D* | -1.585227232 | 1.46E-06 |
| *ANKRD33B* | -1.595963154 | 1.15E-06 |
| *AP000695.1* | -1.627001314 | 2.05E-06 |
| *EPM2AIP1* | -1.634409457 | 5.00E-07 |
| *HIC1* | -1.645856619 | 7.77E-06 |
| *GPR39* | -1.661465805 | 0.000128 |
| *HARBI1* | -1.700897107 | 3.57E-05 |
| *AC025171.1* | -1.731107779 | 3.61E-07 |
| *AC007906.2* | -1.741852006 | 0.000292 |
| *CCDC71* | -1.773416245 | 4.00E-05 |
| *HOXA11* | -1.774539209 | 2.91E-06 |
| *ELMSAN1* | -1.785522503 | 9.16E-09 |
| *HOXA10* | -1.805907693 | 2.45E-08 |
| *APLN* | -1.806464285 | 1.31E-08 |
| *ARL4C* | -1.837834872 | 8.26E-07 |
| *FAM50B* | -1.883085145 | 1.97E-06 |
| *FAM43A* | -1.884613943 | 1.61E-08 |
| *CCDC9B* | -1.983009804 | 9.39E-07 |
| *GIMAP6* | -2.148243871 | 5.19E-09 |
| *FANCF* | -2.197749433 | 2.41E-06 |
| *C1orf74* | -2.916779212 | 1.90E-08 |

**
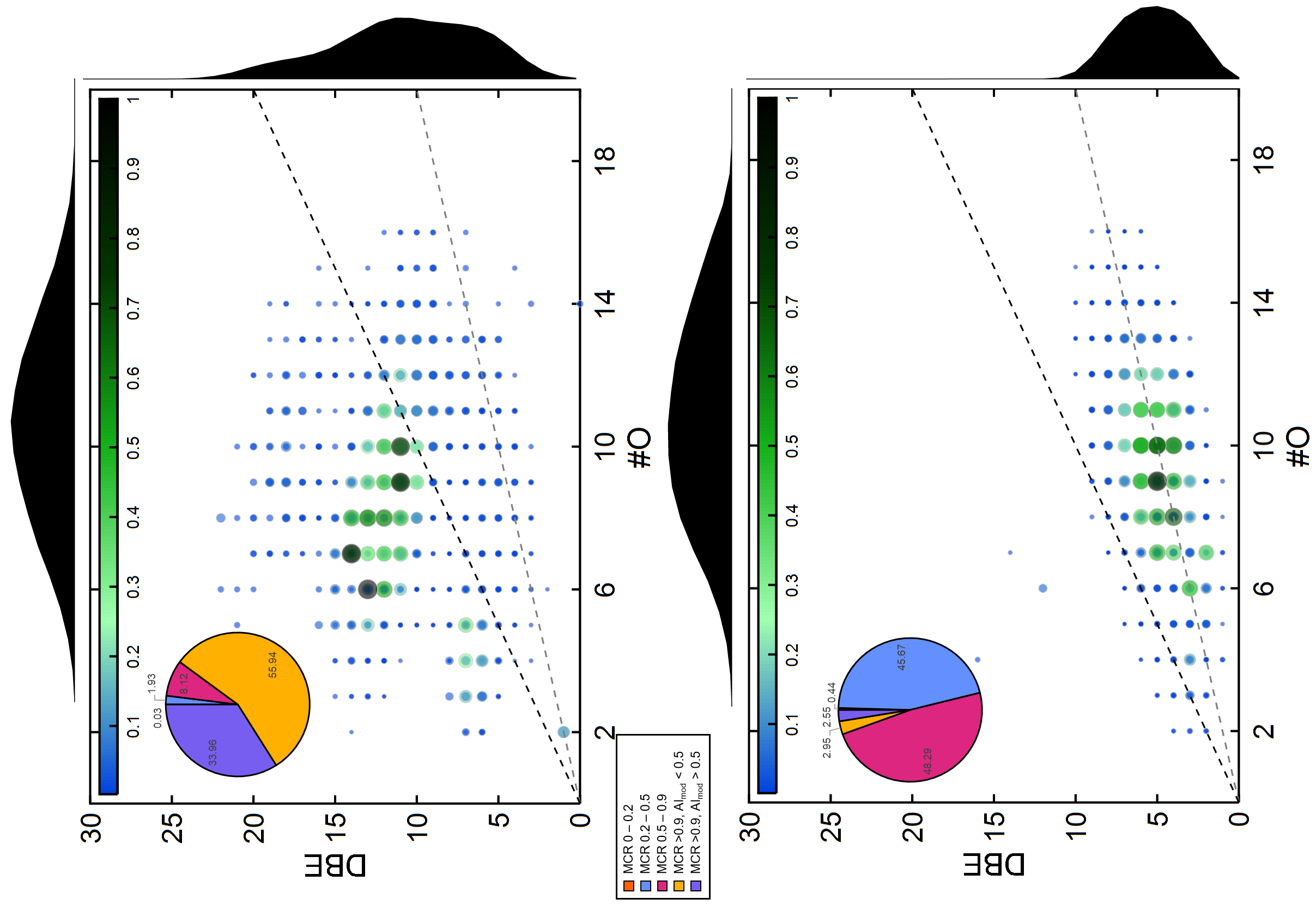
Figure S1.** Number of oxygen atoms (#O) vs double bond equivalents (DBE) in SOA_NAP_-SP (top) and SOA_βPIN_-SP (bottom) with pie charts depicting the percentage of peaks in individual bins of the maximum carbonyl ratio (MCR) and aromaticity index (AI) for ESI in negative mode.

***
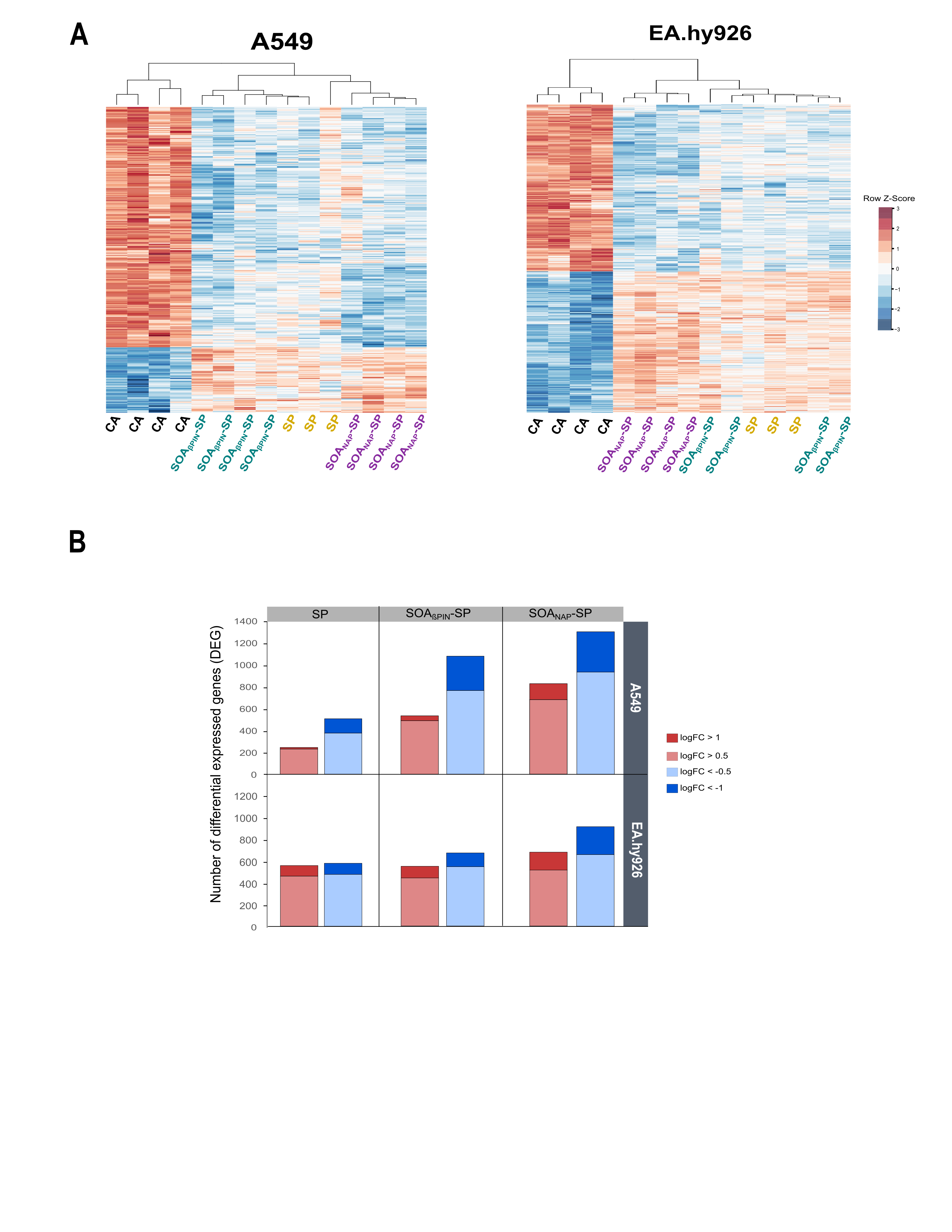
***

**Figure S2. Differential expression analysis.** (**A**) Expression heatmap showing Euclidian sample-to-sample distance matrix with hierarchical clustering of differentially regulated genes in the four exposure groups (CA, SP, SOA_NAP_-SP, and SOA_βPIN_-SP). (**B**) Number of differential expressed genes (DEGs) up- (red) and down-regulated (blue) after the exposure to SP, SOA_βPIN_-SP and SOA_NAP_-SP compared to the CA control in A549 and EA.hy926 cells.

**
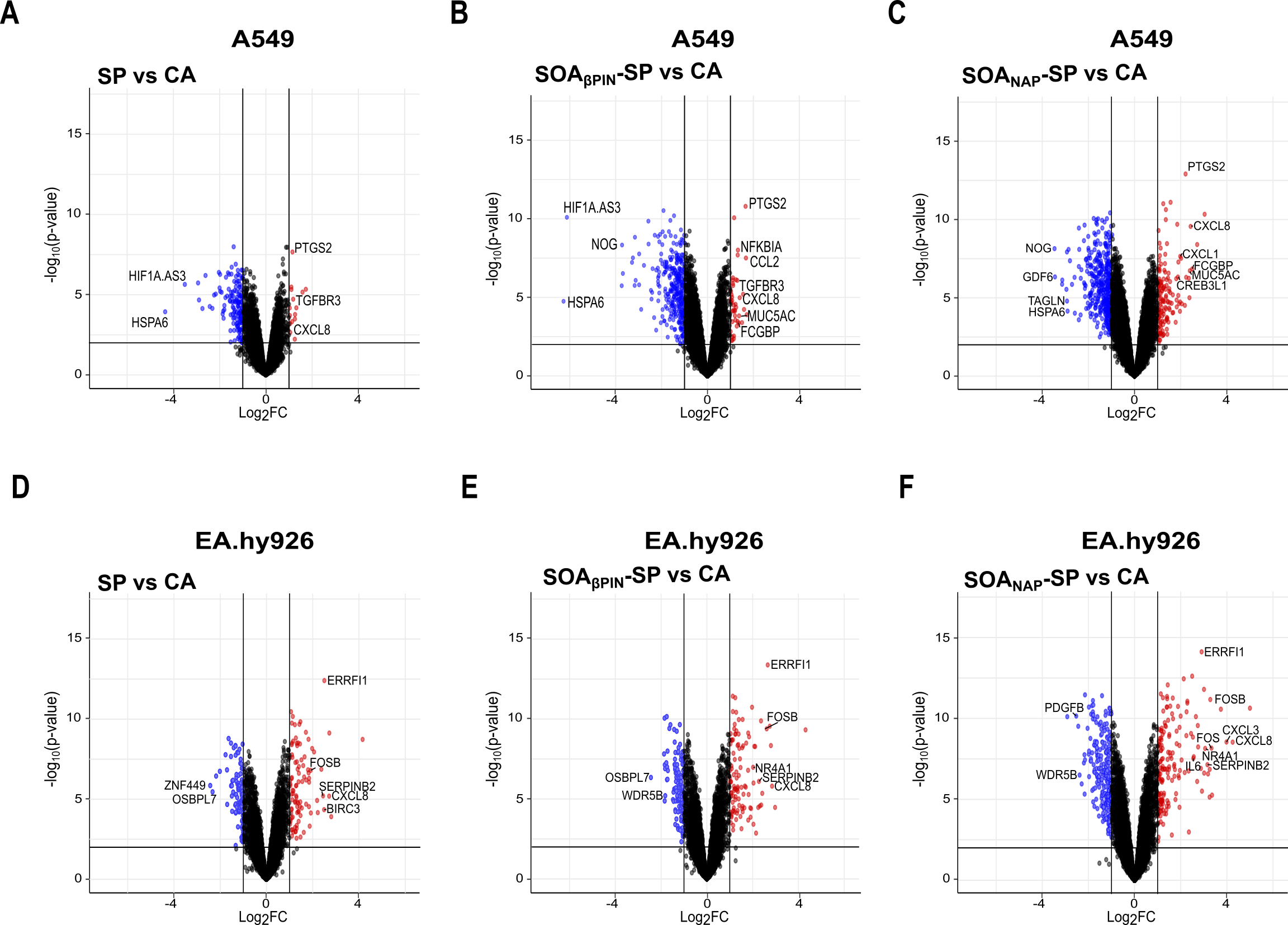
**

**Figure S3. Volcano plots.** (**A - C**) Volcano plots showing the relationship between differential gene expression (adjusted p-value ≤ 0.05 and log_2_FC ≥ 1 and log_2_FC ≤ -1) in A549 after exposure to SP (**A**), SOA_βPIN_-SP (**B**) and SOA_NAP_-SP (**C**). All aerosol exposures are compared to the clean air (CA) control. (**D - F**) Same as in (**A - C**) but based on the analysis in EA.hy926 cells.
